# Sustainability in Health care by Allocating Resources Effectively (SHARE) 6: investigating methods to identify, prioritise, implement and evaluate disinvestment projects in a local healthcare setting

**DOI:** 10.1186/s12913-017-2269-1

**Published:** 2017-05-25

**Authors:** Claire Harris, Kelly Allen, Vanessa Brooke, Tim Dyer, Cara Waller, Richard King, Wayne Ramsey, Duncan Mortimer

**Affiliations:** 1 School of Public Health and Preventive Medicine, Monash University, Melbourne, VIC Australia; 2 Centre for Clinical Effectiveness, Monash Health, Melbourne, VIC Australia; 3 Medicine Program, Monash Health, Melbourne, VIC Australia; 4 Medical Services and Quality, Monash Health, Melbourne, VIC Australia; 5 Centre for Health Economics, Monash University, Melbourne, VIC Australia

**Keywords:** Disinvestment, Decommission, De-adopt, De-list, De-implement, Health technology, TCP, Resource allocation, Decision-making, Implementation

## Abstract

**Background:**

This is the sixth in a series of papers reporting Sustainability in Health care by Allocating Resources Effectively (SHARE) in a local healthcare setting. The SHARE program was established to investigate a systematic, integrated, evidence-based approach to disinvestment within a large Australian health service. This paper describes the methods employed in undertaking pilot disinvestment projects. It draws a number of lessons regarding the strengths and weaknesses of these methods; particularly regarding the crucial first step of identifying targets for disinvestment.

**Methods:**

Literature reviews, survey, interviews, consultation and workshops were used to capture and process the relevant information. A theoretical framework was adapted for evaluation and explication of disinvestment projects, including a taxonomy for the determinants of effectiveness, process of change and outcome measures. Implementation, evaluation and costing plans were developed.

**Results:**

Four literature reviews were completed, surveys were received from 15 external experts, 65 interviews were conducted, 18 senior decision-makers attended a data gathering workshop, 22 experts and local informants were consulted, and four decision-making workshops were undertaken. Mechanisms to identify disinvestment targets and criteria for prioritisation and decision-making were investigated. A catalogue containing 184 evidence-based opportunities for disinvestment and an algorithm to identify disinvestment projects were developed. An Expression of Interest process identified two potential disinvestment projects. Seventeen additional projects were proposed through a non-systematic nomination process. Four of the 19 proposals were selected as pilot projects but only one reached the implementation stage. Factors with potential influence on the outcomes of disinvestment projects are discussed and barriers and enablers in the pilot projects are summarised.

**Conclusion:**

This study provides an in-depth insight into the experience of disinvestment in one local healthcare service. To our knowledge, this is the first paper to report the process of disinvestment from identification, through prioritisation and decision-making, to implementation and evaluation, and finally explication of the processes and outcomes.

**Electronic supplementary material:**

The online version of this article (doi:10.1186/s12913-017-2269-1) contains supplementary material, which is available to authorized users.

## About SHARE

*This is the sixth in a series of papers reporting Sustainability in Health care by Allocating Resources Effectively (SHARE). The SHARE Program is an investigation of concepts, opportunities, methods and implications for evidence-based investment and disinvestment in health technologies and clinical practices in a local healthcare setting. The papers in this series are targeted at clinicians, managers, policy makers, health service researchers and implementation scientists working in this context. This paper reports the exploration of methods to identify health technologies and clinical practices suitable for disinvestment; establish prioritisation and decision-making processes; and develop, implement and evaluate evidence-based disinvestment projects.*

## Background

The need for disinvestment has emerged in response to increasing costs and a growing awareness of ineffective practices and systemic waste in healthcare services. Although there is no clear single definition, disinvestment is generally understood to be removal, reduction or restriction of health technologies and clinical practices (TCPs) that are unsafe or of little benefit, seeking to improve patient outcomes and use available resources more efficiently [1].

Following successful implementation of a rigorous evidence-based program for introduction of new TCPs [2], leaders at Monash Health (previously Southern Health), a large health service network in Melbourne Australia, sought to establish a similar program for disinvestment. However, there is a lack of information to guide local healthcare services regarding an organisational approach to disinvestment [3–12].

The ‘Sustainability in Health care by Allocating Resources Effectively’ (SHARE) Program was established to investigate an organisation-wide, systematic, transparent, integrated, evidence-based approach to disinvestment. The SHARE Program was funded as a 3-year demonstration project by the Victorian Department of Human Services (DHS) and Monash Health, and was undertaken by the Centre for Clinical Effectiveness (CCE), an in-house resource to facilitate Evidence Based Practice (EBP). An overview of the SHARE Program, a guide to the SHARE publications and further details about Monash Health and CCE are provided in the first paper in this series [13].

**Fig. 1:**
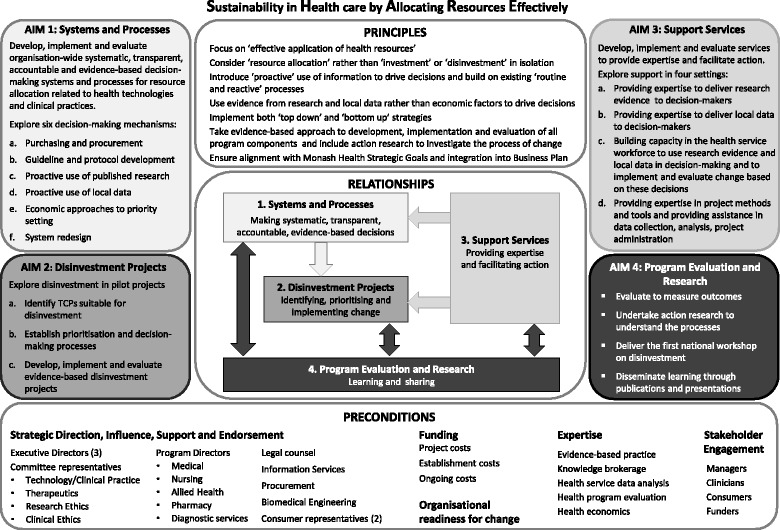
Model for exploring Sustainability in Health care by Allocating Resources Effectively in the local healthcare setting (reproduced from Harris et al. [14] with permission)

Following preliminary investigations to understand the concepts related to disinvestment, identify current decision-making practices at Monash Health, learn from local experiences of disinvestment and consider the implications of the proposed changes, a plan for the SHARE Program was created [14]. This included aims and objectives, principles to underpin the program, preconditions for success and sustainability, and implementation and evaluation plans. The program components and the relationships between them are outlined in Fig. 1.

The first aim of the SHARE Program was to explore systems and processes for decision-making that could proactively and systematically identify opportunities for disinvestment. The second aim was to investigate pilot disinvestment projects to gain detailed insight into the change processes involved, assess the resources required to deliver effective projects, provide practical guidance for future projects and, if successful, be used as positive examples to promote subsequent disinvestment activities.

The preliminary work also identified that if the first two aims were to be achieved, services to support the proposed activities and build staff capacity would be required [14]. Four support services were proposed: an Evidence Service, Data Service, Capacity Building Service and Project Support Service. Piloting of these services became the third aim of the SHARE Program [15, 16].

The need to investigate methods to identify and prioritise potential target TCPs and undertake projects to disinvest them is noted in the literature [5, 9–11, 17–24]. It is also acknowledged that there is little information about implementation of disinvestment decisions, a lack of understanding about the factors that influence resource allocation processes, and under-reporting of the perspectives and experiences of healthcare staff undertaking disinvestment [11, 18, 21–23]. It has been proposed that in-depth research using longitudinal approaches from inception to implementation of disinvestment decisions at the health service level are needed to fill these gaps and contribute to both the theory and practice of disinvestment [18–21]. The fourth aim of the SHARE Program sought to address this.

### Aims

The aim of this aspect of the SHARE Program was to undertake disinvestment pilot projects. This would be achieved via three objectives: identifying potential disinvestment opportunities; establishing prioritisation and decision-making processes; and developing, implementing and evaluating disinvestment projects.

The aim of this paper is to describe, explore and explain the processes and outcomes of undertaking these objectives and the factors that influenced them.

### Research questions

What methods are available to identify potential disinvestment opportunities in a local health service?

What methods are available for prioritisation and decision-making to initiate disinvestment projects in a local health service?

What methods are available to develop, implement and evaluate disinvestment projects in a local health service?

What were the processes and outcomes of application of these methods at Monash Health?

What factors influenced the decisions, processes and outcomes?

## Methods

### Design

#### Case study

The SHARE papers use a case study approach to address the limited understanding of resource allocation processes in health services, particularly regarding disinvestment [18, 21], and the lack of detailed reporting of implementation of change in the literature [25, 26]. Case studies allow in-depth, multi-faceted explorations of complex issues in their real-life settings [27] and facilitate development of theory and interventions [28]. The case study approach enables examination of the complex behaviours of, and relationships among, actors and agencies; and how those relationships influence change [29]. All these issues are intrinsic to the SHARE Program research questions.

All three case study approaches are used [30].

1. Descriptive: findings are reported in detail to describe events, processes and outcomes to enable replication when successful and avoidance or adaptation when unsuccessful
2. Exploratory: literature reviews, surveys, interviews, workshops and consultation with experts are used to explore what is known and identify actual, preferred and ideal practices
3. Explanatory: theoretical frameworks are used to understand and explain the events, processes and outcomes

**Fig. 2:**
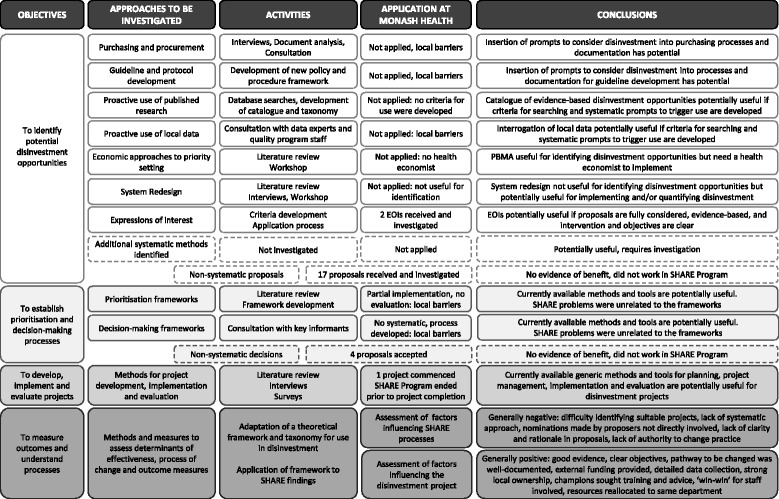
Overview of activities and outcomes

Case studies are characterised by multiple sources of quantitative and qualitative evidence [27]. An overview of the activities undertaken in relation to the objectives is provided in Fig. 2.

#### Model for evidence-based change

The SHARE Program was undertaken using the SEAchange model for Sustainable, Effective and Appropriate change in health services [31]. The model involves four steps: identifying the need for change, developing a proposal to meet the need, implementing the proposal and evaluating the extent and impact of the change. Each step is underpinned by the principles of evidence-based practice to ensure that the best available evidence from research and local data, the experience and expertise of health service staff and the values and perspectives of consumers are taken into account. Sustainability, avoidance of duplication and integration of new processes within existing systems are considered at each step, and an action research component continues throughout the project.

#### Action research

Action research was undertaken based on the ‘researcher as facilitator for change’ model defined by Meyer: researchers working explicitly with and for people rather than undertaking research on them [32, 33]. In this capacity, CCE staff were both the SHARE project team and the action researchers. Observations and reflections of the project team were used for ongoing improvements to the program components and implementation process. An agenda item for ‘Learnings’ was scheduled at the beginning of every team meeting. Participants were invited to consider anything that had affected the project since the last meeting using the framework ‘what worked, what didn’t, why and how it could be improved’. Each issue, its effect on the project, and potential changes that would build on positive outcomes or remove or minimise future problems were discussed. The learnings and actions were documented; actions were assigned, given timeframes and followed up to ensure completion.

#### Development of methods

Several of the activities reported in this paper were to develop methods that would be undertaken in subsequent activities. The methods reported in this section are those determined *a priori*. Methods developed during the course of the investigation are reported in the Results and discussion section.

### Data collection

Mixed methods were used to capture frameworks, methods and tools, and stakeholder perspectives and experiences. These included literature reviews, a survey, interviews, workshops, consultations, and document analysis. Participant validation for factual accuracy was undertaken following interviews and workshops. An overview is provided in Fig. 2 and full details of methods and sources are reported in Additional file 1: Tables A–D.

### Data analysis and synthesis

Outcomes of consultations and findings from initial interviews with small numbers of participants were documented and collated using MS Word or Excel. Workshop and subsequent interview findings were collated in MS Word, Excel and/or Nvivo [34] and analysed thematically by either content analysis [35] to identify emergent themes, or framework analysis [36] when categories had been specified *a priori*. Details of individual project protocols are provided in Additional file 1: Tables A–D.

Using the principles of evidence-based change, the SHARE team worked with stakeholders and external experts to synthesise the findings from the literature and local research into discussion papers and workshop presentations.

### Deliberative process

Decisions were made by the SHARE Steering Committee composed of executive directors, committee chairs, clinical program directors, legal counsel, support service managers and consumer representatives (Additional file 1: Table E). Decision-making workshops were held at scheduled committee meetings. Discussion papers and background documents were provided beforehand, formal presentations introduced the workshops, and topics for discussion and decisions required were listed on the agenda. Discussion was informal within the structure of the agenda and decisions were based on consensus.

### Delivery of disinvestment projects

#### Investigation and selection of proposals

The SHARE team and Monash Health data analysts worked with proposers and the staff members responsible for practice in the nominated areas, usually department heads or committee chairs, to identify relevant research evidence and local data. Findings were presented to Steering Committee members for decision-making.

#### Implementation

Based on the SEAchange model of evidence-based change, planned implementation activities included engaging all stakeholders, identifying what is already known about practice change in the topic area from the literature and local knowledge, undertaking an analysis of local barriers and enablers, developing an implementation plan using strategies to minimise barriers and build on enablers, piloting and revising as required, and implementing in full.

A Capacity Building Service was developed to provide training to the pilot project teams in implementation methods and a Project Support Service was established to provide assistance in project management, administration, ascertainment of barriers and enablers, and development of project plans.

#### Evaluation

An Evaluation Framework and Plan was developed for the overall SHARE Program and included evaluation domains, audience, scope, evaluation questions, outcomes hierarchy, sources of data, methods of collection and analysis, reporting and timelines [37].

Individual evaluation plans for the pilot projects were developed based on the project objectives and an economic evaluation was developed in consultation with the SHARE health economist. Planned activities based on the SEAchange model included evaluation of process (Was the intervention implemented as planned?), impact (Did this achieve a change in practice?) and outcome (Did the practice change address the original problem?). These were not all undertaken due to reduced funding in the final year of the program.

Training in evaluation methods was provided to the pilot project teams through the Capacity Building Service and assistance in data collection and analysis was provided through the Project Support Service.

### Explication of processes and outcomes

**Fig. 3:**
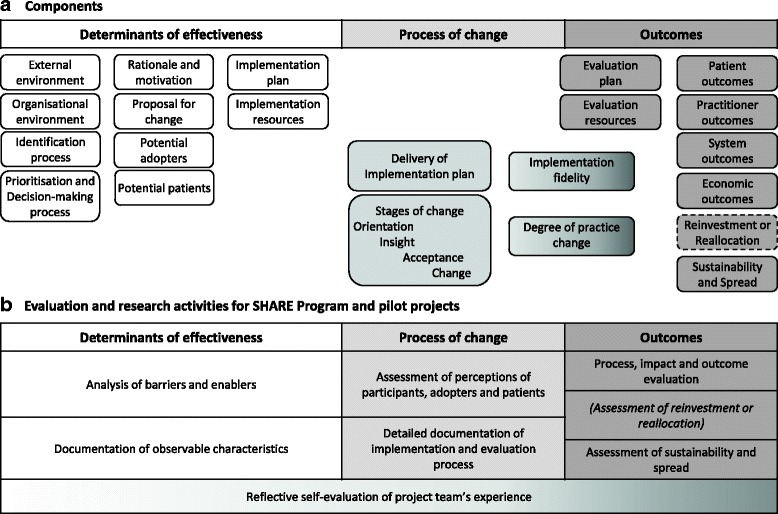
**a**, **b** Framework for evaluation and explication of disinvestment projects (adapted from Harris et al. [163] with permission)

**Fig. 4:**
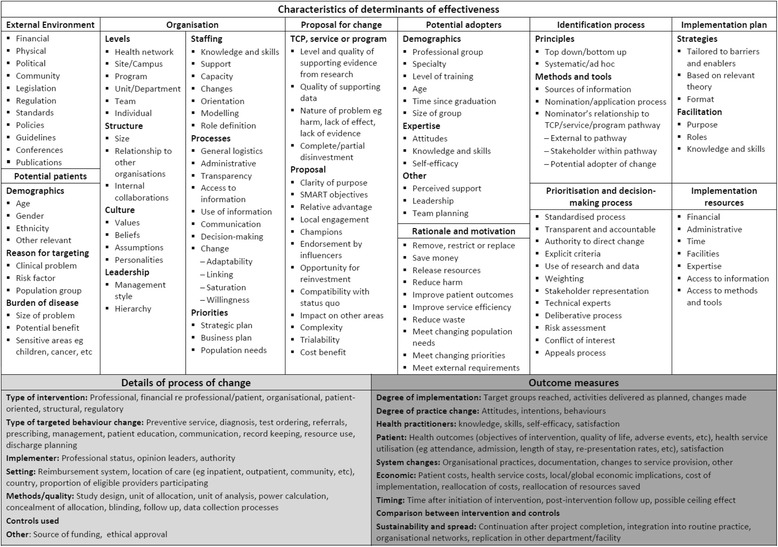
Taxonomy for evaluation and explication of disinvestment project (adapted from Harris et al. [163] with permission)

Factors that influenced outcomes of the piloting process were identified using a framework for evaluation and explication of evidence-based innovations [13]. Based on findings from the literature and surveys and interviews with Monash Health staff, the framework and taxonomy were adapted specifically for use in the context of disinvestment (Figs. 3a and 4). Details of barriers and enablers, observable characteristics of the determinants of effectiveness, perceptions of participants and adopters, the process of change, findings from the action research process and other project team reflections were documented in minutes, reports, spreadsheets and templates for this purpose (Fig. 3b).

## Results and discussion

Some of the planned implementation and evaluation activities were not completed due to reduction of funding in the final year by the program funder and changes in requirements for the pilot project by the project funder; details and impact are discussed below.

Results of the literature reviews and the response rates and representativeness of participants in the survey, interviews and workshops are included in Additional file 1: Tables A–D. Surveys were received from 15 external experts, 65 individuals participated in interviews, 18 senior decision-makers attended a data gathering workshop, 22 experts and local informants were consulted and the members of the SHARE Steering Committee participated in four decision-making workshops.

Data collected from these activities informed a range of research questions. Findings related to the research questions in this paper are presented and discussed below; findings related to topics not addressed here are reported in other SHARE publications [14–16, 38–40].

Although Monash Health staff were not aware of the term ‘disinvestment’, they were familiar with the concept of removal, reduction or restriction of current practices. Surveys and interviews with a range of decision-makers and project staff who had undertaken these and other resource allocation activities provided details of strengths, weaknesses, barriers and enablers in these processes. These have been combined into positive and negative influences to remove duplication; they are collated in Table 1 using the determinants of effectiveness for disinvestment projects (Fig. 3) and discussed within the research questions below.

**Table 1:** Factors influencing resource allocation at Monash Health

Positive	Negative
External environment	
▪ Legislation, regulations, national and international standards, and professional standards must be followed. This provides clarity and certainty for some decisions ▪ International bodies and national agencies of other countries provide evidence-based recommendations for use of health technologies, clinical practices, models of care, etc. Systematic reviews and Health Technology Assessments are also available. ▪ The Australian government provides evidence-based recommendations for use of medical and surgical procedures and drugs ▪ Monitoring, evaluation and reporting of outcomes was required for government funded projects ▪ Department of Treasury is interested in supporting disinvestment initiatives but requires details of savings. If savings or reinvestments can be quantified the department may provide more funding	▪ Some decision-makers are unaware of mandatory requirements ▪ Decision-makers are frequently unaware of evidence-based resources. ▪ Due to lack of time, knowledge and skills decision-makers do not actively seek these resources when making decisions and do not differentiate between high and low quality resources. ▪ Not all medical and surgical procedures and drugs are covered by national policies; nursing and allied health practices, models of care and clinical consumables are not covered ▪ Cost-effectiveness data is often based on modelling which is perceived not to reflect reality ▪ It is hard to measure savings; savings are rarely realised because they are absorbed and used to treat more patients
Organisational environment (Monash Health)	
▪ Enthusiastic and dedicated staff; staff commitment to quality improvement ▪ Organisational support from the Executive Management Team (EMT) and Directors of Nursing ▪ The Board, EMT and Senior Managers have expressed ‘patient-centred care’ as a priority. ▪ Involvement of people who are outside of, or uninterested in, the politics of the organisation ▪ Transparency and accountability in decision-making was highly valued and improved transparency and accountability at Monash Health was desired ▪ At site level there is good ‘buy-in’ for change and people are keen to make things work	▪ Organisational culture is difficult to change ▪ Organisational politics gets in the way ▪ Considerable pressures on the health service to reduce costs. ▪ Lack of processes for project development, implementation, responsibility and accountability ▪ Lack of transparency in all aspects ▪ Lack of transparency and accountability in decision-making reduces confidence; inadequate transparency and accountability was one of the strongest messages ▪ No systematic processes to link projects across the organisation
Identification process	
▪ Projects were identified reactively based on - Government or externally mandated change such as new legislation, regulation or standards; national or state initiatives; and product alerts and recalls. - Clinician or management initiatives arising from awareness of successful projects elsewhere, conference presentations, journals and other publications, and drug and equipment manufacturer promotions. - Problem solving driven by critical incidents, staff or consumer feedback, changing population needs, changing demand for services and budget shortfalls. ▪ Monash Health had well-documented processes for purchasing and procurement and guideline and protocol development and high level expertise in evidence synthesis and utilisation, data analysis and utilisation, and system redesign	▪ General perceptions that - financial drivers stronger than clinical drivers, ‘Sound practice is not always affordable practice’ - impetus for change was ad hoc, there was no systematic or proactive approach - internal bureaucracy and red tape stifled ideas ▪ People by-pass the system and just make changes, usually not deliberate but due to lack of awareness of processes ▪ Some applications for change are driven by pharmaceutical or equipment manufacturers ▪ No examples of using purchasing and procurement, guideline and protocol development, evidence from research or local data, health economic approaches or system redesign to identify potential opportunities for disinvestment were identified
Prioritisation and decision-making process	
▪ Using research evidence and local data in decision making was considered to be important. ▪ All respondents reported using research evidence and data in decision-making to some extent. ▪ Many examples of cross-unit/department consultation and collaboration for policy and protocol development and implementation. ▪ Conflict of Interest was required as a standing item on the agendas of relevant committees. Most committees had a process for conflict of interest for committee members, and some of those with an application process had a similar procedure for applicants.	▪ Only one committee and one individual used explicit, documented decision-making criteria ▪ Only one committee required explicit inclusion of research and local data and considered the quality and applicability of this evidence. Only one of the ten projects appraised the evidence used. The other committees had no process to seek evidence from research. When evidence from research and data was used it was not usually appraised for quality or applicability. ▪ Barriers to using research evidence include no uninterrupted blocks of time, slow computers, lack of skills in finding and analysing evidence ▪ Appropriate local data was frequently reported to be lacking, unavailable and ‘manipulated’ ▪ Decision-making ‘in isolation’, ‘fragmentation’ and a ‘silo mentality’ were reported in relation to decisions made without consideration of the areas they would impact upon or consultation with relevant stakeholders.
Rationale and motivation	
▪ Reasons for previous ‘disinvestment-type’ projects to remove, restrict or replace current practices include reducing patient harm, reducing medication error, reducing unnecessary tests, improving communication, standardising care, saving money and saving time. Most projects had more than one of these objectives	▪ Perceived distinction between ‘what the hospital is concerned about (finances, organisational capacity and risk management) and what the clinician is concerned about (patients)’.
Proposal for change	
▪ When the benefits of the proposed practice change are clear and observable ▪ When there is clarity, relevance, credibility and reliablity of research findings ▪ Availability of quality and timely local data ▪ Sustainability more likely if a range of staff involved, ‘bottom-up’ approaches to change used and monitoring of outcomes undertaken	▪ Lack of baseline data meant that potential adopters were unable to see the benefit or relevance to their situation resulting in less ‘buy in’ and poor uptake.
Potential adopters	
▪ Having the appropriate profession engaging others in change process, for example nurses should be implementing projects with nurses, not pharmacists ▪ Flexible and adaptable staff	▪ Resistance to change ▪ Staff cynicism about the importance of changes and relevance to them ▪ Some clinicians insist on autonomy in their areas of expertise
Potential patients	
▪ Many respondents supported increased consumer participation and were planning to act upon this	▪ Only one committee included consumer representation in decision-making. ▪ Several respondents thought that consumer representation on their committees would be inappropriate or that consumers had insufficient technical understanding to participate.
Implementation plan	
▪ Decisions made at program level that involve multiple wards, departments or sites are usually implemented by multidisciplinary teams ▪ Allowing wards to nominate themselves for participation in projects ▪ ‘Bottom up’ approach to develop individual implementation plan in each ward ▪ Those with project ‘champions’ unanimously considered champions important to the success of the project. ▪ Lots of preparation including training and communication with all stakeholders ▪ ‘Bottom up’ training to gain staff ‘buy in’ combined with ‘top down’ supportive strategy ▪ Training or education included passive methods using posters and memos, interactive learning on new equipment and participatory approaches involving staff in design and implementation.	▪ Things take a long time to implement, to the point that they ‘fall off the agenda’ ▪ Variability in current practice and lack of standardisation increases number of practices to change ▪ Large size, nature and diversity of the organisation increases complexity of implementation across departments with different needsLack of effective implementation pathways ▪ Lack of infrastructure, technical support and resources ▪ High staff turnover in the organisation, particularly agency nurses and junior staff, increases difficulty in communication and implementation ▪ Organisational culture is difficult to change ▪ Organisational politics ▪ High staff turnover in projects diminishes organisational knowledge and expertise and increases training requirements ▪ Competing priorities ▪ Lack of time, undertaking projects while continuing normal clinical duties ▪ One project had no implementation plan ▪ Education and training is not well provided for part-time and night staff
Evaluation plan	
▪ Evaluation and monitoring were considered important and had broad support ▪ Routine clinical audits and monitoring of adverse events undertaken for hospital accreditation purposes provided indirect evaluation of decisions in some situations.	▪ No requirements for evaluation of outcomes of decisions or projects. ▪ Most committees had no planned evaluation of outcomes of decisions or implementation projects. ▪ Quality and Risk Managers are not included at the beginning to help with collection of baseline data and evaluation design
Implementation and evaluation resources	
▪ Finding others who have done the same work for support, advice and information ▪ Establishing Working Parties and Steering Committees for support, endorsement, troubleshooting ▪ Project leader whose primary role is ‘at the coal face’ ▪ CCE was establishing an in-house Evaluation Service at the time of these interviews ▪ Use of pre-existing, pre-tested tools from other organisations eg audit tools ▪ Provision of extra staff ▪ Availability of extra funds enhanced implementation and evaluation, eg introduction of the National Inpatients Medication Chart had external funding specifically for implementation and evaluation ▪ Some clinical pathways involve no additional costs ▪ Some projects were provided with adequate resources for implementation and evaluation ▪ Some wards had additional staffing for education support and clinical nurse support. These were invaluable resources for practice change, protocol development and implementation. ▪ Some projects had external funding from DHS, universities, etc. for staff or infrastructure costs ▪ CCE ran training programs in finding and using evidence, implementation and evaluation ▪ Six of 10 projects had training for project staff in change management, leadership or IT skills.	▪ Unrealistic project timelines ▪ Lack of knowledge, skills and confidence in project management, change management, evaluation methods and tools, and use of information technology. These barriers were exacerbated when interventions were complex and required high levels of training ▪ Lack of/inadequate project management and communication resulted in multiple people making inconsistent changes ▪ Some project staff felt isolated and would have liked support from others who had done the same or similar work ▪ It was not always clear who was responsible for project management ▪ Staffing issues, including leave, mean that a lot of projects are on hold ▪ High staff turnover in projects diminishes organisational knowledge and expertise and increases training requirements ▪ No specified evaluators with appropriate training or expertise had been utilised by the respondents ▪ A lack of data was seen to contribute to the current state of ‘little or no process of evaluation’. ▪ Lack of/inadequate funding, lack of information about available funding ▪ Funding for new equipment frequently did not include funding for training staff to use it or the consumables required. ▪ Many projects were to be carried out ‘within existing resources’. Respondents noted that they either did unpaid overtime or aspects of the project were not undertaken. ▪ Staff dissatisfaction with the expectation of their superiors that they will do more work within existing resources

The investigation of potential methods for identification, prioritisation and decision-making, and implementation and evaluation of disinvestment projects are summarised in Fig. 2. Multiple projects are reported in this paper. To avoid repetition, the Results and discussion sections are combined for each research question.

### What methods are available to identify potential disinvestment opportunities in a local health service? What were the processes and outcomes of application of these methods at Monash Health?

Seven methods to identify disinvestment opportunities in a local health service were investigated. The focus of Aim 1 was to explore methods that could be integrated into organisational infrastructure for systematic consideration of disinvestment in routine health service decisions. Six potential mechanisms were identified (Fig. 1) [38]. Given that it might take some time to identify disinvestment targets from these approaches, a supplementary method was required to find suitable TCPs for immediate implementation in pilot projects in Aim 2. An ‘Expression of Interest’ process was introduced to achieve this.

In addition to the methods noted above, a range of other potential systematic approaches to identify disinvestment opportunities emerged from informal discussions during SHARE activities. These were recorded but not investigated and are listed in Table 2.

**Table 2:** Additional systematic methods to identify potential disinvestment opportunities in a local health service

▪ Consider disinvestment explicitly in long term planning exercises
▪ Discuss principles of disinvestment and examples of successful projects at department/unit meetings, educational events, etc
▪ Assign member of decision-making committees to look for disinvestment opportunities in their decisions
▪ Add a disinvestment question to the Leadership Walkround protocol
▪ Identify clinical champions interested in disinvestment in each program/department/unit who would look out for opportunities
▪ Encourage support staff who have undertaken a disinvestment project to look for more opportunities
▪ Have disinvestment as a high priority in medication safety reviews
▪ Encourage or require projects that are introducing something new to have a component of disinvestment
▪ Review projects that are being conducted for other reasons and identify and focus on any disinvestment elements
▪ Introduce thinking about disinvestment into quality improvement training programs

A non-systematic process of *ad hoc* submissions also emerged during the project and details are reported below.

#### 1. Purchasing and procurement processes

Initial interviews and workshops with key stakeholders identified that systems and processes for purchasing drugs and clinical consumables and capital procurement for building and equipment were potential methods for systematic identification of disinvestment opportunities. Methods to encourage those making decisions about expenditure to consider disinvestment could be integrated into current processes. Prompts, triggers and even mandatory requirements to consider disinvestment could be included in algorithms, protocols, checklists, specific directions within purchase orders, explicit decision-making criteria for committees, or steps in application processes that require authorisation. Incorporating considerations for disinvestment into existing decision-making infrastructure might be achieved quickly and, once established, delivered with no additional costs.

Interviews with staff and analysis of health service documents found that Monash Health had very clear procedures for purchasing but less clear processes for capital expenditure. Only one prompt to consider disinvestment was identified in the wide range of decision-making contexts investigated. The application form for introduction of new TCPs asked applicants to identify current practices that could be discontinued when the new TCP was introduced.

Meetings were held with procurement staff to discuss evidence-based resource allocation processes and consideration of disinvestment. Positive outcomes included participation of the Procurement Manager in the Technology/Clinical Practice Committee (TCPC) meetings regarding introduction of new TCPs, clarification of authorisation processes for new equipment or consumables prior to purchase, and inclusion of a CCE staff member on the Clinical Purchasing Committee to facilitate evidence-based decision-making. However no changes regarding identification of opportunities for disinvestment were implemented. The Purchasing Policy Guidelines were due for routine review and those responsible welcomed participation of the SHARE team to address these issues; however the review was not undertaken during the life of the SHARE Program.

*Discussion*

There are discussions in the current literature about smart, innovative and evidence-based purchasing [41, 42] and the need to consider economic evaluations in purchasing decisions [43], but we were unable to find mention of purchasing or procurement processes being used to identify local disinvestment opportunities.

#### 2. Guideline and protocol development

In addition to processes that allocate funding, systematic mechanisms for allocating non-monetary resources were also recognised by repondents as potential methods to identify disinvestment opportunities. Local guidelines and protocols determine allocation of resources for specific conditions, patient groups or clinical procedures by stipulating use of drugs or equipment, recommending diagnostic tests, selecting health professional groups, prioritising staff time, specifying referral mechanisms and allocating capacity in clinics, operating rooms and other facilities. There are potential opportunities for disinvestment in all of these activities. Prompts, triggers and mandatory requirements to consider disinvestment could be introduced into document development and authorisation processes. Requirements for local guidance to be based on the best available evidence would ensure that harmful, ineffective or inefficient TCPs would be identified in the systematic review process and steps to discontinue these practices could be included in the resulting guidance document. Evaluation, audit and review of guidelines and protocols may also identify opportunities for disinvestment. Mechanisms involving local guidelines and protocols could be implemented quickly and, once established, delivered with no additional costs.

The CCE staff members involved in SHARE were simultaneously developing a new Policy and Procedure Framework for Monash Health. No examples of using local guideline and protocol development to identify disinvestment opportunities were identified from the literature or local consultations in this process.

A prompt to consider whether any current practices could be discontinued was included in the instructions to developers of guidance documents. *“If the procedure involves introduction of new practices, identify the current practices that are being replaced. Cessation or restriction of specific activities in current practice must be addressed with active interventions in the same way as introduction of new practices.”* [44]. A requirement that a systematic review process was followed and a checklist recording the steps undertaken were also included.

After developing the new framework, CCE staff handed it over to the department that had responsibility for organisational documents for implementation and ongoing governance. The disinvestment prompts and requirement for systematic reviews, along with other instructions, were removed by the implementers with the intention of making the process less onerous for document developers.

*Discussion*

Several authors refer to the potential to use guidelines for implementation of disinvestment recommendations [45–49] but we have not found any discussion of local guideline and protocol development being used as a method to identify disinvestment opportunities.

#### 3. Proactive use of published research

Scoping searches of the health databases in preparation for the literature review revealed a growing body of evidence about practices that are harmful, of little or no clinical benefit, or where a more effective or cost-effective alternative is available. Searches for evidence-based disinvestment opportunities could be undertaken and the findings delivered directly to decision-makers. Workshops with the Steering Committee determined that to avoid wasting time and resources considering information that does not represent the best available evidence, only high quality synthesised information such as systematic reviews, health technology assessments and evidence-based guidelines should be used proactively to drive decisions.

**Fig. 5:**
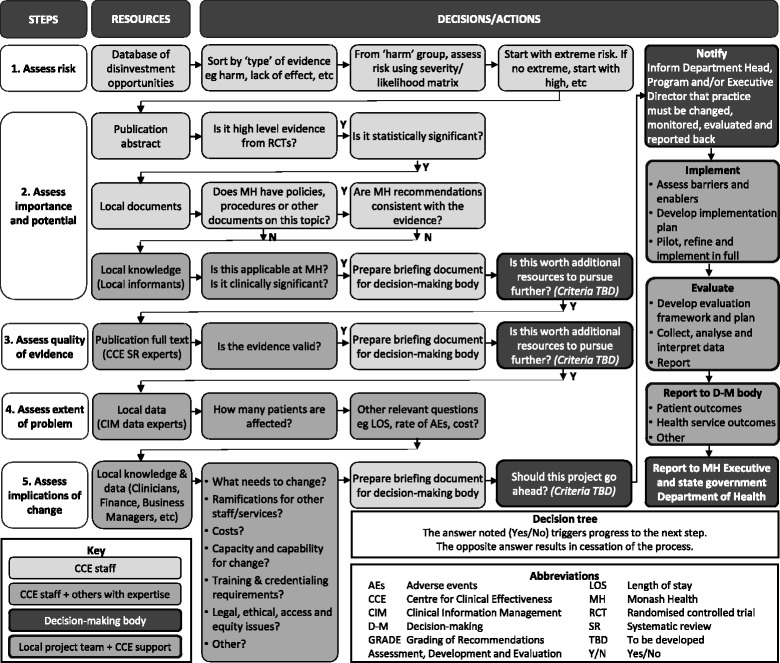
Algorithm for identifying disinvestment projects from an evidence-based catalogue of potential TCPs

It was clear from interviews with decision-makers that Monash Health had no mechanisms to use research evidence proactively. The SHARE team developed a catalogue of disinvestment opportunities to enable this (Additional file 1: Table B). Searches were undertaken in known sources of high quality synthesised evidence to identify TCPs which were demonstrated to be unsafe, not effective or not cost-effective [50–54]. This was supplemented with information from evidence-based publications specifically focusing on disinvestment [55, 56]. A taxonomy was developed to classify publications by Bibliographic Source, Type of technology/practice, Disease group, Age, Gender, Healthcare setting, Professional group, Specialty, Outcomes, Author’s recommendations and Links to original documents. Classifications were based on existing definitions from the National Library of Medicine Medical Subject Headings (MeSH) [57]; International Statistical Classification of Diseases and Related Health Problems, Tenth Revision, Australian Modification (ICD-10-AM) [58]; McMaster Evidence Updates [59]; and Academy Health Glossary of Terms Commonly Used in Health Care [60]. When suitable definitions were unavailable, additional classifications were created and defined to meet Monash Health needs. Potential disinvestment targets were also captured opportunistically by SHARE participants from conferences, journal articles, email bulletins and awareness of practice elsewhere. The project team reviewed research evidence to validate the claims and, if appropriate, add them to the catalogue, bringing the total to 184 TCPs. An algorithm for identifying disinvestment projects from a catalogue of potential TCPs was developed, based on an algorithm previously developed for introduction of new TCPs [2]. To prevent unnecessary resource use, the information is requested in stages, each stage predicated on a positive decision at the stage before (Fig. 5). To minimise the impact on busy clinicians and managers, work that does not require high level skills is undertaken by a project officer. To facilitate objective and trustworthy decisions, work that does require high level skills is undertaken by independent experts proficient in evidence appraisal and analysis of health service data, and transparent criteria are used in deliberation. Local information from policies and procedures, in-house knowledge and experience regarding applicability, and routinely-collected health service utilisation data, are used to inform the decision to proceed with a disinvestment project.

The planned activities were not undertaken. The *ad hoc* approach to identifying disinvestment opportunities discussed below dominated the selection process, leaving no time to develop or apply the proposed systematic methods. The transparent criteria for decision-making were not developed, the catalogue of disinvestment opportunities was not used to identify a potential disinvestment project, and none of the TCPs demonstrated to be harmful, ineffective or inefficient from the research literature were considered by the Steering Committee.

*Discussion*

The concept of a catalogue of disinvestment opportunities has been discussed widely in the literature under the more recently coined term ‘low value’ lists. Lists are being developed by governments and health agencies [55, 61, 62], commissioners of health services [63], professional bodies [47, 64, 65] and researchers [66–68]. Some of these lists are derived from research evidence, some are based on expert opinion and others from a combination of the two. Although removing practices of little or no value clearly has merit, the definition of ‘low value’ is not always explicit and the validity and appropriateness of some of the lists and the ethics of their application have been questioned [67, 69–73]. Duckett and colleagues separate them into ‘top down’ and ‘bottom up’ approaches, noting that each has benefits and drawbacks [74]. The ‘top down’ approaches, such as the UK National Institute for Health and Clinical Excellence ‘Do Not Do’ Recommendations [55], are described as providing the most consistent, objective, transparent and relevant evaluations. The ‘bottom up’ approaches, such as the Choosing Wisely program being replicated in national campaigns across the world [75], highlight potentially ‘low value’ treatments and tests so that clinicians and consumers can consider the relative benefits in their specific situations. Potential users of ‘low value’ lists may wish to confirm the basis for claims made, in particular the definition being used and the use of systematic review evidence in the inclusion process.

#### 4. Proactive use of local data

Respondents in the interviews and workshops to identify potential settings and methods for disinvestment noted that hospitals and other health facilities routinely collect large amounts of data. Three approaches to targeted analysis of routinely-collected data to discover opportunities for disinvestment were identified.

1. To identify areas where disinvestment might have the greatest impact, such as TCPs associated with high volume, high cost, extended length of stay or high rates of adverse events, readmission or re-operation.
2. To investigate variations in practice between organisations, departments and individuals, or over time, that might indicate overuse or inappropriate practices.
3. To explore less commonly used data sources such as complaints registers or patient satisfaction surveys for emerging themes related to inappropriate or undesirable practices.

Interviews with Monash Health decision-makers found that they often used local data to understand problems or develop solutions, but they did not use it proactively to review current practice, seek opportunities for improvement or drive priority setting. While Monash Health was reported to be very responsive to incident reports and complaints on an individual case basis, there were no processes to consider this body of data, seek out patterns or identify areas of concern for further action.

The first two approaches were to be explored within the activities of the proposed Data Service (Aim 3 Fig. 1), but unfortunately this could not be established, mainly due to limited staff capacity and problems with local data access and coordination [15]. The third approach was to be considered in a consumer engagement framework [40], however the incident reporting software and consumer information available from other sources was thought to be inadequate for aggregation and meaningful interpretation, problems that have since been resolved but which prevented exploration at the time. Due to these local barriers, proactive use of health service data was not employed to identify disinvestment targets for pilot projects.

*Discussion*

There is a large body of literature on examination of practice variation [76]. Two recent studies have used practice variation in national and regional settings specifically to identify ineffective practices and note the potential to do so within local health services, or for health services to benchmark against their counterparts [21, 74]. Hollingworth et al. note that many procedures with the highest variability are often not on the ‘low value lists’, indicating additional possibilities to identify disinvestment opportunities from this approach [21]. Use of local data clearly has potential but problems with data validity, reliability, comprehensiveness and degree of sensitivity to disinvestment requirements remain significant barriers [5, 7, 21, 48, 77, 78]. In the same way that the algorithm described above uses local data to substantiate a decision to disinvest a TCP arising from research evidence, research evidence would inform a decision arising from local data by identifying best practice in the relevant area and confirming whether change is needed and what the appropriate alternatives are [38].

#### 5. Economic approaches to priority setting

The literature review exploring the concepts and implications of disinvestment in a local health service found that economic approaches had been used to identify disinvestment opportunities and had potential to do so at Monash Health. Priority setting exercises use economic principles to determine which practices, programs or services to introduce, maintain or remove. Decision-makers weigh up options for investment and disinvestment and select their preferred alternatives using pre-determined criteria established by the stakeholders.

Local respondents were not familiar with health economic methods for priority setting. The subsequent literature review focused on identifying examples of economic methods found two existing reviews that analysed and compared priority setting exercises [79, 80]. Four methods met the criteria of economic analysis applicable at the local health service level; however all of these have limitations in their ability to identify disinvestment opportunities in this context. Health Sector Wide (HsW) Priority Setting, Quality Adjusted Life Year (QALY) league tables and Generalised Cost-Effectiveness Analysis (GCEA) rely on economic evaluation data, making them unsuitable for decisions involving TCPs which do not have any available published economic evaluations [80–82]. GCEA is generally used to make shifts within departmental budgets, rather than across departments or programs [82], also limiting application in the local setting. HsW is designed to shift the focus away from program budgets towards well-defined target populations with particular health problems [81], however health service funding allocation is not based on condition-specific populations. Program Budgeting and Marginal Analysis (PBMA) is the most widely used method; the process is well-tested and guidance is available [79, 83]. It applies the principles of opportunity cost and marginal analysis to determine priorities for health program budgets in the context of limited resources [84]. PBMA has been proposed as a method of ‘rational disinvestment’ [85].

These findings were summarised in a discussion paper and debated at a workshop with the SHARE Steering Committee. Although a health economist had been engaged as a consultant to the SHARE Program, Monash Health had no plans to establish in-house expertise in health economics. The lack of ongoing health economics capability was the key factor in the decision that priority setting exercises were not feasible at Monash Health.

*Discussion*

Although decision-makers acknowledge the usefulness of PBMA, it remains quite difficult to achieve in practice [5, 77, 84]. The major limitations for all priority setting approaches include lack of standardisation in cost-accounting, lack of sufficient high quality data to inform decision-making, and lack of time and skills to undertake the process and implement the decisions [5, 9, 77, 78, 83–85].

#### 6. System redesign

The early scoping searches of the health literature also identified system redesign as another potential method. It is a familiar process in health services and offers a well-accepted context to introduce practice change. System redesign describes a range of methods and tools that have been adapted for use in health care including Lean thinking [86], Clinical process redesign [87], Program Logic mapping [88], Plan Do Study Act quality cycle [89] and Failure Mode Effect Analysis [90]. System redesign could be integrated into a systematic organisational approach to disinvestment.

Information was gathered from another focused literature review to identify examples of system redesign, methods, tools and resources required; and from interviews to investigate system redesign within Monash Health. The literature review was unable to identify examples of system redesign that specifically related to resource allocation decisions for TCPs and, although there was extensive expertise in system redesign at Monash Health, none of the respondents could recall any projects driven by decisions related to resource allocation. However, some of the reported reasons and motivation for system redesign are consistent with principles of disinvestment, for example better use of existing resources, maximising value and eliminating waste, increasing efficiency and reducing duplication of services [91–93]. Monash Health respondents noted that, although disinvestment is not usually an aim of redesign processes, it may be an outcome.

These findings were summarised in a discussion paper and debated at a workshop with the SHARE Steering Committee. The committee decided that system redesign methods would not be used specifically to identify opportunities for disinvestment, but they may be useful in implementing decisions to disinvest and this should be considered for future projects.

*Discussion*

The potential for system redesign as a useful approach to implementing disinvestment has been confirmed in more recent literature [9, 18, 62] and also suggested as a method to quantify disinvestment [62]. Applying the terminology of ‘system redesign’ has also been advocated as a strategy to increase the likelihood of implementation by avoiding the negative connotations of the word ‘disinvestment’ [18, 94].

#### 7. Expression of Interest

A method of rapidly identifying disinvestment opportunities for pilot projects was needed. The Steering Committee proposed that an Expression of Interest (EOI) process where health service staff nominated their own projects could potentially provide quick results.

Monash Health staff were invited to submit applications to receive training and support from the SHARE Program for a disinvestment project. An EOI form was drafted to include criteria agreed by the SHARE Steering Committee. To facilitate completion of the new document, the content and format of existing Monash Health templates familiar to the applicants were adapted to address the EOI requirements. A disinvestment project was defined as one that removes a TCP that is unsafe or ineffective, restricts a TCP to more appropriate patient groups, or replaces a TCP with an equally safe and effective but more cost-effective option. Training in implementation and evaluation methods was provided by the Capacity Building Service. Support available from the Project Support Service included administration; project planning and implementation advice including analysis of barriers and enablers; evaluation advice including establishing systems to monitor and evaluate change and identify sources of data; and economic evaluation or cost comparison study (methodology determined by SHARE health economist). Clinical trials and projects already underway were excluded.

Invitations to submit an EOI were distributed via the Clinical Program Directors. Two applications were received.

*Discussion*

Three more-recently published frameworks for disinvestment also propose applications from stakeholders in the identification process [95–97]; however the effectiveness of this approach has not been established [21, 98].

#### 8. Ad hoc submission process

Many *ad hoc* proposals for potential disinvestment projects were received. At each meeting, members of the Steering Committee nominated TCPs which the SHARE team were asked to investigate. This process was given priority over development of criteria to ascertain suitable TCPs from the catalogue of evidence-based project opportunities. Each proposed TCP had one or more attributes that made it seem promising, but no assessment using explicit criteria was undertaken. Seventeen TCPs were nominated in this way.

Including the two EOIs, 19 TCPs were investigated as potential pilot disinvestment projects. The nature of the change and reason for nomination are summarised in Table 3.

**Table 3:** Potential disinvestment projects

Potential projects and reason for nomination	Source	Result of investigation
1. Reduce ordering of ‘routine’ diagnostic tests in specific setting as thought to be unnecessary and result in increase risk of adverse events and increased costs to hospital and/or patient	Committee member	Not investigated: Further clarification of problem postponed in favour of subsequent proposals
2. Reduce ordering of diagnostic tests in specified setting due to lack of evidence of benefit and concern about validity, reliability and performance of equipment	Committee member	Not investigated: Further clarification of problem postponed in favour of subsequent proposals
3. Reduce ordering of diagnostic tests in specified setting as thought to be of little diagnostic value	Committee member	Not investigated: Further clarification of problem postponed in favour of subsequent proposals
4. Replace equipment with alternative to reduce adverse events and improve patient outcomes in specified patient group resulting in cost savings	Project champion	Not investigated: Project identified too late to be completed within SHARE timelines
5. Replace diagnostic test in specified patient group for one thought to be more appropriate	Committee member	Investigation not completed: Directed by Steering Committee to pursue Therapeutic Equivalence projects
6. Reduce admission of specified patient group as thought to be unnecessary in many cases	Committee member	Investigation not completed: Directed by steering committee to pursue Therapeutic Equivalence projects
7. Replace drug with lower cost but equally effective alternative in appropriate cases as project being undertaken anyway and it would be good way to learn about the change process	Therapeutic Equivalence project	Rejected: Project was already underway
8. Replace drug with lower cost but equally effective alternative in appropriate cases as project being undertaken anyway and it would be good way to learn about the change process	Therapeutic Equivalence project	Rejected: Project was already underway
9. Reduce use of therapeutic intervention due to concerns about safety and effectiveness	Committee member	Rejected: Lack of clarity regarding explicit problem, patient groups, etc.
10. Reduce use of therapeutic intervention as thought to have no evidence of benefit	Committee member	Rejected: Evidence for change unclear
11. Reduce use of therapeutic intervention as thought to have no benefit over less expensive alternative	Committee member	Rejected: Preference to wait until large RCT underway at the time provided conclusive evidence
12. Reduce ordering of ‘routine’ diagnostic tests in specified setting as thought to be unnecessary, result in increase risk of adverse events and increased costs to hospital and/or patient	Committee member	Rejected: Specific setting already planned to be investigated by others in organisational review but timing was unspecified
13. Cease use of therapeutic intervention in specified patient group due to published debate questioning effectiveness	Committee member	Rejected: Evidence not relevant to local patient population
14. Reduce ordering of ‘routine’ diagnostic tests in specified patient group as thought to have no evidence of benefit	Committee member	Rejected: Department could not provide backfill to replace project champion who would undertake project
15. Reduce use of therapeutic intervention in specified patient group due to concerns about patient safety, not recommended in clinical guidelines used elsewhere	Committee member	Decision postponed: While proposer confirmed evidence Rejected: When discovered that project had commenced
16. Replace therapeutic intervention in specified patient group with one considered to be safer, more effective and more cost-effective and funded by state health department	VPACT project	Accepted then Withdrawn: Clinicians became aware of additional evidence and elected to undertake RCT
17. Restrict use of therapeutic intervention in specified patient group as local practice thought to be inconsistent with recently published national guidelines	Expression of interest	Accepted then Withdrawn: Clinicians not convinced by evidence, local practice found not to be inconsistent
18. Reduce ordering of diagnostic tests considered to be inappropriate in certain unspecified situations	Expression of interest	Accepted then Rejected: Inopportune timing due to external accreditation process and introduction of new computer database and electronic ordering system
19. Replace therapeutic intervention in specified patient group with one considered to be safer, more effective and more cost-effective and funded by state health department	VPACT project	Accepted: Project undertaken with SHARE support but evaluation incomplete due to loss of funding prior to completion of implementation

*Discussion*

Proposals based on individual’s observations or local knowledge have been referred to as “*soft intelligence*” [21]; this has been described in attempts at disinvestment by others and noted to be unsustainable [21, 23, 99].

### What methods are available for prioritisation and decision-making to initiate disinvestment projects in a local health service? What were the processes and outcomes of application of these methods at Monash Health?

#### Prioritisation framework and tool

The priority setting exercises described above clearly include a prioritisation process, however initiatives that identify disinvestment targets by other means may need a specific prioritisation process to choose between the available options.

A literature review to identify frameworks and tools for prioritisation found a Spanish guideline and assessment tool specifically for disinvestment [100], a framework for priority setting in the Australian context [101, 102], a guidance document for prioritisation of new or existing technologies [103], and two systematic reviews and an overview of international practice in prioritisation of new technologies [104–106]. Consultation with local informants identified that replacement of high cost medical equipment had to meet the requirements of the state government Medical Equipment Asset Management Framework (MEAMF). Interviews with local decision-makers identified that there were no decision-making settings at Monash Health where disinvestment was explicitly considered, hence nowhere to pilot prioritisation tools. The Steering Committee directed the SHARE team to develop a tool that could apply to both investment and disinvestment and pilot it in the annual capital expenditure funding round.

The Australian priority setting framework [101, 102] was adapted for use as a local template and the Spanish PriTec prioritisation tool [100] was modified to address MEAMF requirements and include relevant elements from the TCPC application forms [2]. Equivalent criteria for comparison of non-clinical technologies such as information technology and building works were developed as they are considered alongside health technologies in the capital expenditure process. The tool included methods of establishing criteria, a suite of domains from which criteria could be selected, potential questions that can be asked within each domain, scoring systems, processes for weighting criteria and a template to record decisions. These were workshopped with the Steering Committee and members of the Capital Expenditure Committee and refined based on their feedback. The tool was not tested; the capital expenditure process was cancelled in that year as Monash Health had no spare capital.

*Discussion*

Subsequently, lists of criteria for consideration in prioritisation and decision-making have been published for disinvestment [22, 107–110], resource allocation [111, 112] and general decision-making [113], and software applications are now available to facilitate prioritisation processes [83, 114]. Other more recent publications have noted that, like Monash Health, most decision-makers use their own prioritisation matrix based on simple spreadsheets or business case templates and that this variety of tools makes it difficult to compare costs and outcomes within and between agencies [9, 77, 94].

#### Decision-making to proceed with a disinvestment project

Prioritisation tools primarily focus on characteristics intrinsic to the TCP. However additional criteria may influence whether a TCP is selected to be the focus of a practice change initiative. These might be factors that affect the outcome of a project such as likelihood of success or sustainability and potential usefulness of the evaluation, or pragmatic features that enhance initiatives chosen specifically as pilot or demonstration projects such as opportunities for ‘quick wins’.

Criteria for the EOI process were developed based on information from the literature and stakeholder consultations, and refined in consultation with the SHARE Steering Committee. The EOI criteria stipulated that the project must be based on high-quality evidence, be endorsed by Program and Department Heads, have appropriate resources allocated to undertake the project, have a documented clinical pathway and clear measurable outcomes. These and additional criteria that emerged in general discussion during SHARE meetings are outlined in Table 4. However no explicit decision-making criteria were established to prioritise or make final decisions regarding pilot projects.

**Table 4:** Examples of criteria for selection of disinvestment projects considered in the SHARE Program

Criteria in the SHARE Expression of Interest application
▪ The project must aim to remove, restrict or replace a technology or clinical practice ▪ There must be high-quality evidence for the proposed change (as indicated by existing systematic review or body of evidence from peer reviewed articles) ▪ Department and Program heads endorse the proposed change ▪ Department or Program agrees to provide EFT/project leader to implement the proposed change ▪ The current clinical pathway is documented or a commitment is given to document this pathway before the project begins ▪ There are clear, measurable outcomes and ability to collect baseline and comparison data
Criteria that may increase the likelihood of project success or sustainability
▪ Project leaders who have the power to make change happen in their area of responsibility such as Unit Managers or Department Heads ▪ Project champions who are respected and trusted by the potential adopters ▪ Interested, engaged clinicians working in the topic area ▪ Available funding ▪ Projects that propose reallocation of resource savings
Criteria that may be useful for selection of pilot or demonstration projects in disinvestment
▪ Projects that are already planned for another reason that also contain an element of disinvestment ▪ Projects to introduce a new TCP where disinvestment of an existing practice can be made a focus of the project ▪ Opportunity for a ‘quick win’
Criteria that may increase the usefulness of a pilot or demonstration projects in disinvestment
▪ Projects that are required to collect detailed data, for example reporting requirements of external funders ▪ Projects with robust data at baseline

The decisions made were pragmatic, based on likelihood of ‘quick wins’ and unspecified factors related to the proposed TCP. Prioritisation did occur, but the reasoning was not transparent. The final outcomes and reasons for the decisions are summarised in Table 3. Of the 19 proposed TCPs, four were not investigated as the Steering Committee directed the SHARE team to disregard them in favour of subsequent proposals which were thought to have greater potential; two had incomplete investigations for the same reason; and nine were rejected for a range of issues. Four applications were accepted. The first was withdrawn almost immediately by the clinical project leaders who became aware of additional evidence that reduced their confidence in the original decision and elected to undertake a randomised controlled trial (RCT) instead. The second had moved into the development and planning phase when the clinical project leaders initially questioned the evidence underpinning the guideline recommendation they were implementing, and subsequently decided that the practice to be disinvested was not routinely performed at Monash Health. The third had potential as a disinvestment activity but was not well defined. The SHARE team worked with the clinical project leaders to identify and quantify the problem and clarify the proposed practice change; however the project was withdrawn when it became clear that external factors would prevent it from being achieved within the original SHARE timelines (this decision was made prior to reduction of funding in the final year of the program). The fourth project went ahead. Two of the four projects accepted were from the EOI process and the other two had external funding from the Victorian Policy Advisory Committee on Technology (VPACT). VPACT funding was provided to implement new technologies, however both projects had an element of disinvestment as the new TCPs were replacing a clearly identified current practice.

*Discussion*

Deciding between several alternatives can be a complex process requiring consideration of multiple factors. This has been addressed in more recently developed tools. Multi-criteria decision analysis (MCDA) allows consideration of all factors simultaneously [115, 116] and Accountability for Reasonableness (A4R) is based on four principles ensuring that decisions are relevant, transparent and able to be enforced and appealed [117]. MCDA is the foundation for the Star model (socio-technical allocation of resources) [118–120] and the EVIDEM framework (Evidence and Value: Impact on DEcision Making) [121]; both of which have been piloted, revised and produced resources to aid implementation. A4R is the basis for the 6-STEPPPs tool (Systematic Tool for Evaluating Pharmaceutical Products for Public Funding Decisions) [122] and A4R and MCDA have been combined in other decision-making applications [115, 123].

### What methods are available to develop, implement and evaluate disinvestment projects in a local health service? What were the processes and outcomes of application of these methods at Monash Health?

The initial literature review and survey of external experts did not identify any information to guide development, implementation or evaluation of disinvestment projects in the local health service context. Interviews and workshops with Monash Health staff found that, although they did not use the specific term, they had experience of ‘disinvestment’ processes and other resource allocation activities. Most of the issues they identified (Table 1) were consistent with well-recognised factors such as the effect of organisational culture, value of stakeholder involvement, and lack of time, skills and resources. Others were less well-known such as unrealistic project timelines, the importance of support from colleagues who had done similar work, and lack of organisational processes for project development, implementation, evaluation and governance. Respondents also identified needs for assistance including capacity-building, provision of expertise, practical support tailored to needs of individual units and health professional groups, and incentives for change.

Only one of the proposed pilot disinvestment projects reached the implementation stage (Table 3). Nursing and allied health staff were introducing a non-surgical technique in a subset of patients currently being treated with a surgical procedure. The surgeons were happy to relinquish these cases to reduce the waiting time for their other patients.

The clinical project team attended workshops on evidence-based change, implementation and evaluation and worked with SHARE staff to develop project, implementation, evaluation, reporting and cost-comparison plans. The funding agency required Monash Health to include four other health services in this project but no additional time or resources were provided. Many of the activities in the planning and development phase of the project were not undertaken as this time was spent liaising with the other health services. Analysis of barriers and enablers was delayed until midway through the implementation process which precluded development of strategies to avoid or minimise problems before they arose; however identifying actual, rather than anticipated, influencing factors provides more accurate information for future use (Table 5). The Project Support Service provided assistance in identifying indicators to meet reporting requirements; designing and developing a data collection tool and purpose-built database; training in data entry and analysis; liaising with data providers, statisticians and the SHARE health economist; and ongoing problem solving.

**Table 5:** Factors influencing the SHARE pilot disinvestment project

Positive	Negative
External environment	
▪ The project funders had significant impact on the project - Political support for new technology ▪ The other health services in the consortium also had significant impact - Collaboration with some of the other health services in writing pathway and documents and developing database and implementation strategies was helpful ▪ Manufacturer’s information was useful ▪ Manufacturer’s technical representative was helpful	▪ The project funders had significant impact on the project - Monash Health informed that they had to lead a consortium of health services in implementing the new technology, adding complexity to the original application - Lack of consultation in choice of partner health services - Requirements for data collection and reporting changed during the project ▪ The other health services in the consortium also had significant impact - Slow and difficult to coordinate when working with other health services - Lack of accountability in some of the other health services - Lack of ‘buy-in’ from other health services through the entire process
Organisational environment (Monash Health)	
▪ Monash Health’s reputation as a leader will facilitate new technology support ▪ Monash Health encourages innovation ▪ Support from Centre for Clinical Effectiveness (CCE) ▪ Support from Clinical Program Directors ▪ Support from Finance Department and having someone who can translate the finance jargon ▪ Clinical Resource Nurse monthly meetings ▪ Nursing/Allied Health collaboration ▪ Although staff leave and secondments are difficult there can also be an advantage of working with replacement staff who become familiar with the project	▪ Organisational processes appear to be changing regularly ▪ Lack of clarity around organisational structures and processes eg who to go to for what, when etc. ▪ Lack of communication eg machine delivered to a corridor on a Friday afternoon and left unsecured over the weekend. A component was lost and a new component had to be purchased. ▪ Relevant patient group and clinical expertise in this area located at site A and new machine is at site B. Patients usually scheduled for surgery at A will have to transfer to B. ▪ Sites have different cultures and processes and patients and staff will have to adapt ▪ Impact on other departments eg Sterilisation department has to learn new procedure ▪ Staff secondments and/or leave
Identification process (VPACT application process for introduction of new TCP)	
▪ Proposed by potential adopters (nursing/allied health and surgeons) ▪ Support from CCE to provide supporting evidence ▪ Support from Clinical Information Management to provide supporting data	▪ Application form is really long and a lot of work ▪ Lack of awareness of the workload prior to commencing work on application
Prioritisation and decision-making process (SHARE process to determine disinvestment project)	
▪ VPACT funding and endorsement ▪ Clinical project team keen to access CCE expertise and support for project delivery	
Rationale and motivation	
▪ To reduce harm, improve patient outcomes, improve service efficiency, save money	▪ Emphasis on financial/economic outcomes
Proposal for change	
▪ There is good evidence to support the new technology ▪ Data on patient group, burden of disease, impact of new technology provided in detail ▪ New technology does not cause long lasting/irreversible damage ▪ Easy to use ▪ Proposal for change is clear ▪ Relative advantage is clear: improved outcomes for both patients and health service ▪ Endorsed by clinical leaders, good local engagement, clinical champions ▪ Surgeons allowed to keep the theatre time and reduce their own waiting lists (rather than reallocating to other surgical specialties or closing theatres to realise savings)	▪ Longer time to set up than other treatment options ▪ Lots of protective clothing which can be uncomfortable ▪ Mentally and physically tiring ▪ The whole process of change including administration, training, support, etc. is a lot of work
Potential adopters (Nursing and Allied Health staff to undertake new procedure, surgeons to reduce old procedure, junior medical staff to refer patients appropriately	
▪ Most surgeons happy to relinquish old procedure to allow them to undertake other procedures ▪ Surgeons involved in VPACT application have become an authority on the new technology ▪ Senior clinical staff read up on new technology as they don’t want to lose face ▪ Registrars (referrers) are supportive of/have an interest in new technologies ▪ General interest among staff ▪ Nursing/Allied Health team look professional, able to build credibility and trust with patients	▪ One group of surgeons less likely to refer patients for new procedure, do not appreciate role of podiatrist in patient care, lack of understanding of treatment options ▪ Some surgeons/medical staff have issues with territorialism and ego
Potential patients	
▪ Patients with chronic conditions are more open to trying new treatments	▪ This group of patients are less likely to be comfortable travelling to different hospitals ▪ Lack of English language can be a problem
Implementation plan	
▪ Small training workshops with medical teams ▪ Support from CCE ▪ Support from Clinical Program Directors ▪ Maintenance of a booking system ▪ Quarterly meetings with all participating health services	▪ Should have performed barriers and enablers analysis earlier in process ▪ Involvement of other hospitals with staff who are not dedicated/committed (eg disputes among doctors from another site) ▪ Having to repeat training every 3–6 months due to staff rotations ▪ Attrition of podiatrists and Clinical Nurse Consultants as they are often young women who leave or work part-time to have or care for children ▪ Keeping the team motivated is hard ▪ VPACT did not meet costs stipulated in application; fewer machines, limited consumables, etc. ▪ Lack of dedicated treatment room increases time for preparation and cleaning. Clinical time is small in comparison to set up/clean up time. Inadequate ventilation (aerosols are created with treatments)
Evaluation plan	
▪ Support from CCE in development of evaluation plan ▪ Having a person in charge of data entry	▪ ‘Shifting the goal posts’ by VPACT regarding data collection and reporting
Implementation and evaluation resources	
▪ Other clinical staff voluntarily take up extra workload (both barrier and enabler) ▪ Support from CCE in design of a database, assistance with data entry and reporting ▪ Support from SHARE health economist in development of cost-comparison plan ▪ Monash Health ‘Scope of practice’ processes and documents were helpful	▪ Inadequate funding for clinical staff to implement and evaluate change process ▪ Other clinical staff voluntarily take up extra workload (both barrier and enabler) ▪ Time needed to write up new scope of practice documents

As the SHARE Program concluded earlier than expected, the implementation phase had not been completed and the planned evaluation was not undertaken. While we understand that the new technology was implemented and the transition from the old procedure to the new procedure was generally successful, final outcomes were not measured. The clinical project team agreed to complete the same template used by the SHARE project team to capture their experiences: ‘what worked, what didn’t work, how could it be improved?’ There is considerable overlap between these findings and the barriers and enablers. They have been combined and collated under the headings of the determinants of effectiveness in Table 5. Many of these are context-specific relating to the clinical procedure, requirements of the funding body, and relationships between stakeholders; however others identify issues likely to be common to local healthcare settings such as impact on other departments, difficulties moving between sites or finding new clinical accommodation, and one health professional group not accepting the role of another. The benefits of in-house expertise and support provided for development, implementation and evaluation were highlighted.

*Discussion*

The current literature acknowledges generic needs for implementation strategies and methods for monitoring and evaluation of disinvestment outcomes. In concert with the responses from Monash Health staff, several authors call for dedicated resources and in-house “*resource centres*” to provide expertise, access to relevant methods and tools, and education, training and capacity-building [9, 11, 95, 124, 125]. A guideline for disinvestment details eight steps in an Action Plan [96], some authors note principles for implementation and others discuss barriers and enablers [98]. A range of theoretical approaches to facilitate implementation of disinvestment decisions has been proposed but the authors do not report application or evaluation of these strategies in the disinvestment context. These include communication and educational materials [6, 7, 63, 70, 107, 109]; financial incentives and pay-for-performance [46, 70, 109, 126, 127]; reinvestment of resources saved [8, 18, 107, 128]; clinical champions [18, 77]; clinical pharmacists to monitor and advise prescribers [129]; quality standards [70, 127]; professional standards, maintenance-of-certification activities and practice audit [70]; prompts through guidelines, protocols, clinical pathways and decision support systems [6, 7, 24, 48, 109, 126, 127]; requirements to report variations from mandatory guidelines [127]; monitoring and reporting of outcomes [107, 109, 126]; public reporting of provider performance [70, 109, 126, 127]; training and re-organisation of staffing and equipment [107]; and “*picking low hanging fruit*” before tackling more difficult projects [18]. The Schmidt framework for disinvestment notes that both process and outcome evaluations should be undertaken but provides no other details [95]. Others propose measures for both procedure aspects and outcomes in priority setting projects [130] and list evaluation tools linked to specific project/program goals [131]. A systematic review summarises a range of performance measures to assess use of low value TCPs [132]. The deficiencies in available economic and usage data and lack of methods for quantifying savings are considered to be significant limitations to evaluation [11, 24, 48, 78, 133].

### What factors influenced the decisions, processes and outcomes?

The factors identified in relation to the determinants of effectiveness are summarised in Table 5 (pilot project) and Table 6 (SHARE process). Due to the shortened timelines our ability to draw conclusions is limited, but we can describe and discuss key findings related to process and impact in the context of known influencing factors from the current literature.

**Table 6:** Factors influencing the SHARE process of selecting disinvestment projects

Positive	Negative
External environment	
▪ The SHARE program was adequately funded (until the final phase of the program) ▪ Two proposals that received state health department funding and endorsement were considered favourably. ▪ Two proposals were triggered by new national guidelines, one by an editorial in the Medical Journal of Australia, and others by journal articles, email bulletins, attendance at conferences and proposers awareness of practice elsewhere.	▪ The state health department withdrew funding for the final phase of the SHARE program resulting in reduction of the proposed evaluation activities. ▪ One project was rejected due to difficulties implementing change during the national accreditation process for this department’s services.
Organisational environment (Monash Health)	
▪ Monash Health encourages and supports innovation ▪ High level expertise was available from CCE and Clinical Information Management	▪ Waiting for responses to email correspondence and requests for appointments to meet with key personnel; time lags due to annual and long service leave and decisions by committees that only meet monthly delayed the processes of identification, prioritisation, decision-making and project development. Delays in deciding that unsuitable projects would not go ahead prevented other potentially suitable projects from being investigated. ▪ The proposer of one project was unaware of an existing organisational review into the problem. ▪ Delays related to introduction of a new computer database and electronic ordering system contributed to one project being rejected.
Identification process	
▪ The ‘bottom up’ Expression of Interest process was the only systematic approach used, resulting in two projects being received and accepted (but both later rejected).	▪ The ‘top down’ evidence-based catalogue of disinvestment opportunities was not utilised in identifying potential projects. ▪ The ‘ad hoc’ process of nominations and decision-making dominated ▪ Most proposals were made by ‘outsiders’ not involved in the nominated clinical pathway. Only two proposals were made by the potential adopters, although one subsequently withdrew their application.
Prioritisation and decision-making process	
▪ All discussions were held within meetings and documented in the minutes; there were no attempts to be covert or follow hidden agendas. ▪ Conflict of interest was addressed as a routine agenda item. ▪ All clinical programs, health professional disciplines, consumers and technical experts in evidence, data, legal, ethics, finance, purchasing, biomedical engineering and information technology were represented in decision-making.	▪ There were no explicit processes for risk assessment, deliberation or appeal. It was not always clear how decisions had been made. ▪ The SHARE Steering Committee did not have authority to direct change. Proposals were put to department heads who declined to follow them up (based on reasoned arguments that they should not to go ahead).
Rationale and motivation	
▪ Safety and effectiveness were the primary reasons for nominating TCPs for disinvestment, cost-savings were a secondary benefit	
Proposal for change	
▪ Six proposals were submitted based on guidelines, systematic reviews or health technology assessments; the four accepted projects were in this group. ▪ Four proposals had supporting data, two regarding unnecessary diagnostic imaging tests and the two VPACT projects. ▪ The two VPACT projects presented defined objectives. ▪ One project had a clear reinvestment plan which allowed operating theatre time previously used by patients now undergoing the new non-surgical procedure to be used by other patients on the waiting lists, this was the implemented pilot project.	▪ In 13 proposals, the nominator did not provide supporting evidence. ▪ Many of the proposals did not clearly define the TCP, patient population group, circumstances of restriction, etc. This is difficult to quantify as clarification may have been forthcoming but the proposals were not investigated further
Potential adopters	
▪ Three nominations were made by the potential adopters; one was the pilot project accepted and implemented, one was accepted as a pilot project but was subsequently withdrawn by the applicants and the other was nominated too late to be included in the SHARE timeframe	▪ Decisions regarding eight proposals were declined by heads of the departments responsible for the proposed TCP. Reasons included lack of clarity of the problem, lack of supporting evidence, or the evidence was not relevant to local patient groups. ▪ In two of the accepted projects, the key adopters reversed their decisions about the supporting evidence and withdrew.
Potential patients	
	▪ Two proposals were rejected when it became clear that the evidence did not apply to the Monash Health population.
Implementation and evaluation plans and resources	
▪ The CCE/SHARE support staff had appropriate expertise and knowledge of methods and tools for implementation and evaluation. ▪ The CCE team provided access to research literature and liaised on behalf of the clinical project teams with the Clinical Information Management (CIM) unit who were happy to provide access to data and assistance with analysis. ▪ All implementation activities within the control of the SHARE project team were completed ▪ Detailed evaluation plans were developed in consultation with an external health program evaluator and health economist ▪ One proposal had assistance of a research fellow to undertake the project work (but this did not go ahead for other reasons). ▪ The clinical project leads of two accepted projects attended workshops in evidence-based change, implementation and evaluation	▪ Lack of evaluation funding precluded understanding of the barriers that prevented implementation of the planned systematic evidence-based processes ▪ Lack of evaluation funding limited evaluation activities in the last year of the program ▪ One project was rejected by the department head because they could not provide backfill for the clinical duties of the project leader.

#### Difficulty identifying disinvestment projects

The challenges in identifying suitable disinvestment projects are well documented. Decision-makers find it difficult to identify appropriate disinvestment opportunities [5], even when provided with evidence-based lists of appropriate options [48, 134]. Having made a decision, they are often uncertain about whether it is correct [5] and some prefer to avoid the decision and *“invest to save”* as an alternative to disinvestment [18]. Decision-makers can be enthusiastic supporters of disinvestment in theory, but unable to select TCPs for disinvestment in practice [21].

The experiences at Monash Health are consistent with these. Only one suitable project emerged from 19 nominations. Three factors played a significant role in this lack of success: dominance of an *ad hoc* process to select targets for disinvestment, local barriers beyond the scope of the SHARE Program, and lack of clarity and substance in proposals for change. These are discussed below.

#### Non-systematic approach

The absence of standardised methods for disinvestment decision-making is well-recognised [11, 18, 19, 23, 99]. Lack of transparency was reported in the earlier explorations of decision-making at Monash Health [39] and is also discussed in the literature in relation to disinvestment processes [7, 23, 62, 77, 83, 99, 135].

*Ad hoc* approaches to disinvestment decisions have been reported as *“non-sustainable, reliant on chance or not conducive to independently identifying local opportunities for disinvestment”* [21], compromising transparency and leading to uncertainty [23]. The gap between rhetoric and reality is described as the heart of the challenge related to disinvestment in healthcare policy and practice [99]. The experience that *“a lot of decisions are taken on gut feeling”* and the problematic *“tendency to adopt a short term perspective whilst searching for a ‘quick fix’ instead of taking a whole systems perspective based on consideration of long-term sustainability”* [99] reflects the SHARE experience.

Although the SHARE Program was underpinned by a commitment to systematic, transparent, accountable and evidence-based systems and processes, this was not achieved in the process of delivering pilot disinvestment projects. Potential target TCPs in the evidence-based catalogue were not considered and nominations were accepted and pursued in an *ad hoc* manner.

SHARE had all the recognised enablers to systematic use of synthesised evidence in decision-making [136–140]. The decision-makers understood the usefulness of systematic reviews, the program was committed to EBP, and the organisational culture was supportive. The CCE team had the appropriate skills and were sufficiently resourced to identify and access the evidence, ensure its applicability, highlight the relevant message and deliver it directly to decision-makers. Yet the planned systematic approach using synthesised evidence was not followed. The shortened timelines prevented exploration of the reasons for this unexpected outcome.

The non-systematic approach also led to a lack of transparency. All discussions were documented in minutes of the meetings and there were no attempts to be covert, however in the absence of a specified process and explicit criteria, it was not always clear how decisions had been made. The decisions themselves were transparent but the methods to reach them were not.

There were four exceptions to the *ad hoc* approach: two projects were based on a systematic, explicit EOI process and two had been through a rigorous application process for VPACT funding. These were the four projects finally accepted.

#### Nominations by ‘outsiders’

*“Understanding how the technology got on the agenda, where it came from and who was pushing for it”* have been reported as important factors for senior health decision-makers [135]. When invited to nominate candidates for disinvestment, clinicians frequently identified the practices of other professional groups rather than their own [21, 70].

This is also true of the SHARE process. Eight proposals were made by people who had no connection with the TCP pathway. In addition, two were proposed because they were proceeding anyway (Therapeutic Equivalence Program) and two were proposed by the state health department unit (VPACT) providing funding to implement new TCPs (Table 3). In total, 12 were proposed by ‘outsiders’. Five proposers were participants in the TCP pathway but were not the clinicians whose practice was nominated for change. Only three nominations were made by the potential adopters; one was the pilot project accepted and implemented, one was accepted as a pilot project but was subsequently withdrawn by the applicants and the other was nominated too late to be included in the SHARE timeframe.

#### Authority and ownership

Noted barriers to EBP include lack of authority to make the change [78, 84, 137, 139–142] and lack of ownership by key stakeholders [84, 143–145].

Most of the SHARE activities were either within the remit of CCE or the portfolios of the executives and senior managers on the Steering Committee. However the SHARE team did not have ownership of the data services, purchasing and procurement processes, and guideline and protocol documentation, or authority to make decisions in these departments. Although managers in these areas were generally supportive, their heavy workloads and competing priorities unrelated to SHARE activities prevented successful implementation of change in these areas.

#### Rationale and motivation

Disinvestment has been associated with a perceived focus on ‘cost cutting’ and ‘taking away’ in preference to ‘evidence-based care’ [21, 23, 62, 146], even to the extent that alternative terms have been introduced to avoid this [18, 62]. Improving the quality of care while reducing costs is one of the key arguments for ‘value for money’ achieved through disinvestment, highlighting the tension created by the implication that health services can deliver better care while saving money [48, 62, 134, 147–149].

Monash Health staff also perceived that “*financial drivers were stronger than clinical drivers*” in previous decision-making processes (Table 1).

In contrast, this was not a notable feature in the SHARE process. Only two projects were explicitly initiated to save money; the Therapeutic Equivalence process aimed to replace high cost drugs with lower cost but equally effective alternatives. These projects were included as potential pilot projects as they were already going ahead. All nominations arising directly from the SHARE process related to safety and effectiveness of the drugs, clinical procedures or diagnostic tests proposed for disinvestment. In five cases, cost-savings to the hospital and/or patients was noted as a secondary outcome arising from reduced adverse events or improved patient outcomes. Although disinvestment of most of the proposed TCPs was likely to result in cost-savings this was not mentioned as a priority in the nomination or decision-making processes.

Eleven proposals were to reduce use of a TCP, six were to replace an existing TCP with a better alternative, one was to restrict practice in a defined patient population and one was to cease practice altogether. Seven proposals were for inappropriate or overuse of diagnostic tests.

#### Proposal for change

Clarity of aims and objectives and a clear proposal for change are significant factors in successful disinvestment [99].

Lack of clarity in the proposal for change is the reason that proposed TCPs did not proceed to guidance for disinvestment; specific issues include insufficient information on the population, intervention, comparators and outcomes; harms and benefits not clearly summarised; evidence that the intervention was effective or promising for some groups, and therefore potentially not ‘low-value’ for all patients; variation in the conclusions reached in similar scenarios; and uncertainty due to a lack of evidence, low quality or no evidence, and lack of clinical or statistical significance [134].

These findings are very similar to the SHARE experience. Only four of the proposals clearly defined the TCP, patient population, clinical indications and supporting evidence at the time of nomination. Three went on to be accepted as pilot projects and the fourth was discovered not to be applicable in the Monash Health context. Of the 13 proposals investigated, five were rejected or withdrawn due to insufficient evidence to support the proposed change (Table 3).

The pilot project was the exception, with many favourable factors in the proposal for change (Table 7). Proposals are more likely to be successful if they have certain characteristics [150–152] and new initiatives are more likely to be sustainable if there is appropriate and adequate provision of critical factors to achieve and maintain the proposed components and activities [153]. These characteristics are summarised in the checklist for success and sustainability used in the SHARE Program [14]. The factors that make a project likely to be successful as a disinvestment initiative in a local health service are unknown, however the pilot project had many factors considered favourable by decision-makers in the SHARE Program (Table 4). In particular, there was good evidence of better patient and health service outcomes, strong local ownership and clinical champions, a ‘win-win’ scenario for adopters where nursing and allied health staff were keen to take on new procedural skills and surgeons were happy to relinquish these cases to make operating theatre time available for other patients, and surgeons were allowed to keep the theatre time and reduce their own waiting lists (rather than reallocation to other surgical specialties or closing theatres to realise savings).

**Table 7:** Factors for success, sustainability and suitability for disinvestment in the SHARE pilot project

SUCCESS
A proposal is more likely to be successful if it meets the following criteria
Based on sound evidence or expert consensus
✓ Systematic review of multiple RCTs; surgeons, nurses and allied health staff in agreement with findings
Presented by credible organisation
✓ Review undertaken by the Australian Safety and Efficiency Register of New Interventional Procedures – Surgical (Royal Australasian College of Surgeons)
Able to be tested and adapted
✗ *There was limited opportunity to test and adapt as the VPACT funding required complete roll out*
Relative advantage is evident
✓ Clear evidence of multiple improved patient and health service outcomes; increased safety and effectiveness, reduced costs
Low complexity
✓ The new technology is easy to use
Compatible with status quo
✓ Referrers use the same referral process but divide patients into those eligible for the new procedure and those who should still undergo the old procedure
✗ *The new service was provided at a different campus and patients and staff had to adapt*
✗ *There is some impact on other departments that also have to adapt*
Attractive and accessible format
✓ The new procedure is attractive to patients as it replaces surgery with an outpatient/bedside procedure
SUSTAINABILITY
A proposal is more likely to be sustainable if it has appropriate and adequate provision in each category
Structure
✓ The new procedure is carried out within existing nursing and allied health structures with appropriate governance and supports
Skills
✓ Nursing and allied health staff were upskilled in the new procedure; changes in scope of practice were documented and approved
✓ Clinical project team leaders attended training and welcomed support and direction in project management, implementation and evaluation
Resources
✓ Funding was provided for staffing, equipment and consumables
✗ *Final funding was less than the amount approved in the application process leaving the project short of one machine and associated consumables*
✓ Assistance from the Capacity Building and Project Support Services was provided
Commitment
✓ The project had organisational commitment from the Technology/Clinical Practice Committee, and program and departmental commitment from clinical leaders and managers
Leadership
✓ The clinical project team demonstrated effective leadership
SUITABILITY FOR DISINVESTMENT
Factors in the pilot project considered likely to be favourable for a disinvestment project at Monash Health
✓ The current practice to be replaced and the new practice to be implemented were clear and patient eligibility was determined
✓ The proposal for change was clear with clear objectives
✓ Department and Program heads endorsed the change
✓ External funding was available
✓ The clinical pathway and referral process were documented
✓ Detailed data collection and reporting was a requirement of the external funding
✓ Baseline data had been collected and supporting data on patient group, burden of disease and impact of the new technology was available
✓ There was strong local ownership and clinical champions
✓ ‘Win-win’ scenario for adopters where nursing and allied health staff were keen to take on new procedural skills and surgeons were happy to relinquish these cases to make operating theatre time available for other patients
✓ Surgeons were allowed to keep the theatre time released by the changes and reduce their own waiting lists (rather than reallocation to other surgical specialties or closing theatres to realise savings)
✓ Potential ‘quick win’ scenario for a disinvestment demonstration project as the proposal was already fully developed, funding had been approved, and deadlines were in place.
Key: ✓ Positive factors ✗ Negative factors

#### Provision of support

Lack of knowledge and skills in project management, implementation and evaluation and lack of time to carry out the related activities are widely recognised as barriers to effective change in health care generally and resource allocation in particular [5, 7, 9, 18, 43, 77, 84, 95, 96, 124, 125, 135, 139, 154]. Dedicated resources and in-house “*resource centres*” have been proposed as potential solutions [9, 11, 95, 124, 125, 155, 156]. These findings were confirmed in local surveys and interviews at Monash Health [15, 39].

To address these issues, the SHARE Program implemented services to provide expertise and support to decision-makers and project teams [15]. A Capacity Building Service provided training in implementation and evaluation methods and a Project Support Service provided assistance in project management and delivery. All aspects of these support services were valued highly by participants.

### Limitations

The findings come from one organisation and there may be many differences with other health services which limit generalisability. However many of the results are similar to existing reports.

Funding was reduced in the final year of the program; hence the pilot project was not fully implemented and some of the planned evaluation activities were not completed when the program concluded, limiting our ability to draw conclusions based on final outcomes.

Several of the nominated projects were not fully investigated prior to being rejected; so we can comment on factors that were noted in these cases but cannot say that factors we did not observe were not present.

The project team responsible for delivering the SHARE Program at Monash Health were also the researchers investigating the processes undertaken. This has the potential to introduce subjectivity into the evaluations and limit insight if organisational assumptions are accepted without challenge. Detailed exploration and documentation of ‘learnings’ throughout the project, extensive stakeholder involvement, transparency of methods and participation of an external evaluator in the role of ‘critical friend’ [14] were included in the SHARE processes to minimise these limitations.

### Contribution of this study

This study provides an in-depth insight into the experience of a systematic approach to disinvestment in one local health service. To our knowledge, it is the first paper to report the process of disinvestment from identification, through prioritisation and decision-making, to implementation and evaluation, and finally explication of the positive and negative factors influencing the processes and outcomes in a local healthcare setting. This contributes in part to addressing the acknowledged gaps in the current literature [5, 9–11, 18–21].

A range of novel methods not previously discussed in the disinvestment literature were identified and investigated. They provide a range of ‘top down’ directive approaches and ‘bottom up’ invitation strategies.

This study also addresses the lack of models and frameworks noted in the disinvestment literature [4, 5, 8, 10, 11, 19, 149, 157–159]. Firstly, a framework and taxonomy for evaluation and explication of implementation of change have been adapted specifically for use in disinvestment projects. They were used to describe, explore and explain the characteristics of the determinants of effectiveness that influenced the process and outcomes and identify potential influencing factors that have not previously been reported in the context of disinvestment. Secondly, methods to create an evidence-based catalogue of disinvestment opportunities and an algorithm to identify potential projects from the catalogue have been developed.

### Implications for policy and practice

The main messages from this paper may be about ‘what not to do’.

Firstly, seeking out targets with the specific aim ‘to disinvest’ did not work in the SHARE Program, or as reported by others [5, 18, 48, 77, 134]. There are many specific challenges to the concept of disinvestment that may account for this [1]. Although we were unable to capture the stakeholder’s perspectives of the processes used to identify TCPs suitable for disinvestment, we know from previous work at Monash Health and the literature in this area that the word ‘disinvestment’ is associated with negative connotations, risk of engendering suspicion and distrust, and getting stakeholders offside [7, 14, 62, 146, 157, 160]. Yet successful removal, reduction or restriction of healthcare practices and services are commonplace. In these cases the impetus for change is not ‘to disinvest’ but to meet more constructive aims such as to improve patient safety, implement evidence-based practices, address changing population needs or redirect resources to more pressing priorities [39]. In fact, the only successful SHARE disinvestment project was one that aimed to introduce a new technology; disinvestment was only a component of the change process, not the purpose of the project.

Secondly, if health service decision-makers seek to identify TCPs that are not safe, effective or cost-effective (rather than seeking ‘to disinvest’), an *ad hoc* process of accepting proposals may not be the most effective approach. It did not work here, or as reported by others [21, 70, 99]. There is a lack of information about effective systematic methods, however the seven approaches discussed above and other methods identified but not explored hold potential.

There are also positive messages from this work. Although the objective to deliver disinvestment pilot projects was largely unsuccessful, there is much to learn from these experiences and the findings contribute in part to addressing the paucity of information about the disinvestment process. The single project undertaken was underpinned by a rich list of enabling factors, also contributing to the knowledge base in this area.

It has been argued, within the SHARE Program and by others, that disinvestment would be more successful when considered in conjunction with investment decisions [1, 14, 85, 161]. Principles for a decision-making program [98] and incentives for more effective disinvestment [161] have been proposed in this context.

### Implications for research

While it may not be productive to specifically seek ‘to disinvest’, it is appropriate and worthwhile to remove practices that are harmful, ineffective and inefficient. There are many potential sources of information and decision-making mechanisms to identify these practices. The opportunities for research lie in development of proactive methods and systematic prompts and triggers to utilise these resources.

Seven potential methods of identifying disinvestment opportunities were investigated. While system redesign and PBMA were not feasible as methods of identifying disinvestment targets at Monash Health, both approaches are now well-researched, including their role in disinvestment [9, 18, 62, 83–85, 94]. The other five methods still hold promise and, to our knowledge, have not been explored elsewhere. Since local factors were responsible for their lack of success in the SHARE Program, further investigation of the potential within existing health service infrastructure for purchasing and procurement systems and guideline and protocol development to identify disinvestment opportunities, and development of new processes to drive disinvestment decisions proactively with evidence from research and local data or proposals from health service stakeholders is warranted. In other situations, or with other methods of investigation and implementation, they may prove to be effective tools.

The framework and taxonomy for evaluation and explication of disinvestment projects, and the algorithm for identifying disinvestment projects from a catalogue of potential TCPs, can be tested and refined for use in this context or extended into other decision-making settings.

## Conclusion

Local barriers were responsible for the limited success in applying the novel methods in this project. Further exploration of proactive methods to identify suitable disinvestment targets, systematic prompts and triggers to initiate disinvestment decisions, and strategies for project development, implementation and evaluation is warranted. Detailed documentation of the processes undertaken and the factors influencing them provide insight into elements to build upon and others to be avoided in future investigation of disinvestment in the local healthcare setting.

## Additional file

Additional file 1: Methods. (PDF 888 kb)

## Abbreviations

A4R: Accountability for Reasonableness
CCE: Centre for Clinical Effectiveness
CGEA: Generalised Cost-Effectiveness Analysis
EOI: Expression of Interest
EVIDEM: Evidence and Value: Impact on DEcision Making
HsW: Health Sector Wide
MCDA: Multi-criteria decision analysis
MEAMF: Medical Equipment Asset Management Framework
NICE: National Institute of Health and Clinical Excellence
PBMA: Program Budgeting and Marginal Analysis
QALY: Quality Adjusted Life Year
RCT: Randomised controlled trial
SHARE: Sustainability in Health care by Allocating Resources Effectively
STEPPP: Systematic Tool for Evaluating Pharmaceutical Products for Public Funding Decisions
TCP: Technology or clinical practice
TCPC: Technology/Clinical Practice Committee
VPACT: Victorian Policy Advisory Committee on Technology
