# Supplementary material for: Sustainability in Health care by Allocating Resources Effectively (SHARE) 6: investigating methods to identify, prioritise, implement and evaluate disinvestment projects in a local healthcare setting
Source: BMC Health Serv Res. 2017 May 25;17:370. doi: 10.1186/s12913-017-2269-1 (PMC5445482; doi:10.1186/s12913-017-2269-1)
Supplement: Additional file 1: Methods. — (PDF 888 kb) [file 12913_2017_2269_MOESM1_ESM.pdf]

Sustainability in Health care by Allocating Resources Effectively (SHARE) 6: Investigating methods to identify, prioritise, implement and evaluate disinvestment projects in a local healthcare setting

**Additional file 1: Methods**

## Contents

Table S1. Identification of potential methods ........ 2

Table S2. Investigation of methods for identification of disinvestment opportunities at Monash Health ........ 4

Table S3. Investigation of methods for prioritisation and decision-making at Monash Health ........ 8

Table S4. Investigation of methods for implementation and evaluation at Monash Health ........ 8

Table S5. Deliberative process ........ 9

Table S6. Interview schedule System Redesign ........ 9

References ........ 10

## Table S1. Identification of potential methods

| |
|---|
| **Literature review**<br>**Aim:** To understand the concepts related to disinvestment and their implications in a local health service and to ascertain examples of existing decision-making systems and processes in this setting.<br>**Search terms:** Medical Subject Headings (Health Care Rationing, Resource Allocation, Health Priorities and Health Services Needs and Demand) and Text words (disinvestment, decommissioning, defunding, resource release, allocation, reallocation, hit list, ineffective services, low value services, wish list, exclusions, priority setting, program budget marginal analysis, PBMA, resource scarcity, rationing, invest to save) were used with truncations appropriate to the databases utilised. The search strategy was iterative with new terms added as they were identified.<br>**Sources:** Medical databases (Ovid Medline, All EBM Reviews, EMBASE, Cochrane Library), the internet (via the Google search engine) and guideline websites. These methods were supplemented by follow up of reference lists in key publications and searches for publications by identified authors in the field.<br>**Inclusion criteria:** English language publications including guidelines, reviews, research studies, technical reports or policy documents that addressed the issue of disinvestment from a conceptual (terminology, definitions and operational criteria) or policy perspective.<br>**Data Collection and Analysis:** Inclusion, exclusion and appraisal criteria were established a priori. Publications that did not meet the criteria were excluded on review of title and abstract. When a decision could not be made based on abstract alone, full text was retrieved. Critical appraisal appropriate to study design was planned but no research studies were identified.<br>**Search results:** Nineteen documents met the inclusion criteria. These were mainly publications providing a statement of the policy context, the rationale or need for disinvestment and/or a critique of existing processes. A small number of case reports were included but no research studies were identified.<br>**Synthesis:** Information from articles which met the inclusion criteria was summarised based on content relevant to the themes of conceptual and policy perspectives determined a priori.<br>Full details are in the review publication [1]. |
| **Interviews with members of the Technology/Clinical Practice Committee**<br>**Aim:** To identify opportunities for disinvestment in existing or potential decision-making settings and consider implications for disinvestment in the Monash Health setting<br>**Participants:** The Executive of the Technology/Clinical Practice Committee (TCPC), the initiators of the SHARE Program, included an executive director, medical director, clinical program director and research director.<br>**Data collection:** Semi-structured group and individual discussions were conducted using prompts based on the two aims; discussions were documented in the minutes.<br>**Collation:** Responses were collated and added to findings from the other sources which were then analysed thematically by content analysis.<br>**Response rate:** All 4 informants participated.<br>**Representativeness of sample:** Participants represented senior decision-makers from a range of contexts |
| **Survey of external experts**<br>**Aim:** To ascertain unpublished experiences or examples of models or methods for disinvestment in the local healthcare setting.<br>**Participants:** 1) Disinvestment researchers initially identified from publications and websites about disinvestment and subsequently using a snowballing technique based on feedback from respondents. 2) Subscribers to the Health Technology Assessment (HTA) email list.<br>**Design:** The organisation-wide systematic approach to disinvestment proposed in the SHARE Program was described in an email. Participants were asked if they had experiences of disinvestment in the local healthcare context that could inform Monash Health decision-making, any unpublished reports or other documents on this topic, and current or planned research in this area.<br>**Data collection:** Responses were received by return email.<br>**Analysis:** Responses were collated and added to findings from the other sources which were then analysed thematically by content analysis.<br>**Response rate:** Eleven of the 14 researchers and four health librarians from the HTA list (denominator unknown) responded to the survey. |

**Structured workshops with the SHARE Steering Committee**

**Aim:** The workshops had several aims, the component reported in this paper relates to identification of opportunities for disinvestment in existing or potential decision-making settings.

**Inclusion criteria:** Senior decision-makers at Executive and Director level and health service consumers

**Sampling:** Convenience sampling was used to include members of the SHARE Steering Committee comprising Executive Directors (Medical, Nursing, Support Services), clinical Program Directors (Medical, Nursing, Allied Health, Pharmacy, Diagnostic Services), Committee chairs (Technology/Clinical Practice, Therapeutics, Human Research and Ethics, Clinical Ethics), Directors of non-clinical services (Information Services, Clinical Information Services, Procurement, Biomedical Engineering, Research Services), Legal counsel and two consumer representatives. Two representatives from the Department of Human Services Technology Division also participated.

**Approach:** Workshops were conducted at scheduled Steering Committee meetings.

**Design:** Workshops were based on the first two steps in the SEAchange model for evidence-based change [2]; identifying the need for change and developing a proposal for change. Presentations outlining the background and aims of the workshops were made by the project team, discussion was structured around the questions to be addressed and decisions were based on consensus. Questions included:

*Workshop 1:* Where and how are decisions made, documented, communicated, implemented and evaluated and what are the related system issues? Where is change required? Why? What is the problem? How can the need for change be measured? What are the factors enabling sustainability of the current system? How is it integrated?

*Workshop 2:* What existing systems/processes work well that we could maintain as they are, should be ceased, could be kept but require improvement? What new systems/processes should be introduced? What structures, skills, resources, commitment and leadership are required? Are they available? If not, how can they be obtained? What existing systems can be utilised? What is the solution to the problem? What are the options? What is known about best practice in this area? What is required to ensure sustainability of the proposed system? How can it be integrated?

**Data collection:** Participants completed prepared worksheets and discussed the findings. Discussion and decisions were documented in minutes.

**Respondent validation:** Minutes were approved at the following meeting.

**Analysis:** Data from the worksheets and findings from the discussion were collated and organised in MS Word and Excel. Emergent themes were identified by framework analysis.

**Response rate:** Thirteen members participated, 9 attended the first workshop, 11 attended the second, and some non-attenders also completed the worksheets.

**Representativeness of sample:** A range of senior decision-makers were represented at each workshop, plus representatives from the state health department.

**Interviews with key local informants**

**Aim:** To test preliminary thoughts regarding direction of the SHARE Program with front line staff and consumers

**Participants:** Six participants selected purposefully and pragmatically to seek the views of a range of Monash Health decision-makers: the five senior clinicians were program directors and department heads representing medicine, surgery, nursing, allied health and diagnostic services and the consumer representative had experience on committees that made organisation-wide decisions.

**Interview schedule**

*Disinvestment*: Have you heard about the concept of disinvestment?

*Potential settings/methods*: Are you aware of any of these? Do you do any of these sorts of things? What could you do in your Unit? What could be done in your Program/Division? What could be done by your colleagues eg referrers? Any opportunities for quick wins? Incentives to change? Barriers to change? Potential to link into advanced trainee projects?

*Research evidence*: What information do you use? Where from? How do you access it? What do you do with it? Could you use more? What would you like? How would you like it? What would you do with it?

*Local data*: Do you use Monash Health data? How? What for? Do you use external data? What? How? What for? Could you use it? How?

*General discussion*: How could we get wider feedback? Should we survey, etc? Should this be driven top down or bottom up? Would you be interested in piloting something?

**Data collection:** Structured interviews were conducted using the interview schedule above; one CCE staff member attended and took notes.

**Analysis:** Responses were collated and added to findings from the other sources which were then analysed thematically by content analysis.

**Response rate:** All 6 informants participated.

**Representativeness of sample:** Interviewees represented senior decision-makers from a range of contexts

## Table S2. Investigation of methods for identification of disinvestment opportunities at Monash Health

### 1. Purchasing and procurement

**Structured interviews with staff authorised to make decisions on behalf of the organisation**

**Aim:** To identify and document current processes for making, implementing and evaluating decisions and the factors that influence them.

**Inclusion criteria:** Staff and consumers authorised to make decisions regarding resource allocation for health technologies and clinical practices at organisation-wide level in group or individual settings.

**Sampling:** Purposive and snowball sampling was used.

- Twenty-two committees were initially identified from a governance structure diagram. A further 20 were identified through a snowballing method by asking participants in the subsequent interview process, senior managers and Quality Unit staff if they were aware of others. Fourteen of the 42 potential committees met the inclusion criteria (Capital Expenditure, Falls Prevention, Information Systems Governance, Joint Program Quality and Safety, Medication Safety, Operating Suite Product Evaluation, Nurse Standardisation of Practice, Resuscitation, Skin Integrity and Pressure Ulcer, Sterilising Services, Technology and Clinical Practice, Therapeutics and Transfusion Committees and the Executive Management Team).
- Approved Purchasing Units (APUs) have delegated authority from the Board to commit the organisation to a legal and/or financial obligation such as issuing a purchase order or signing a contract. Of the nine APUs, two had been included in the group decision-making committees (Capital Expenditure Committee and Executive Management Team) and five others met the inclusion criteria (Pharmacy, Health Technology Services, Equipment Services, Procurement and Clinical Purchasing, and Materials Management).
- Clinical managers from one clinical program selected for its high use of health technologies were identified from the program's intranet page. Individuals were selected purposively to represent all levels within the program's decision-making hierarchy; medical and surgical sub-specialties, nursing and quality management; and a range of campuses.

**Approach:** Personalised email invitations from the project team were sent to the Chair, Executive Sponsor and/or Secretary of 14 committees, managers of 5 APUs and 9 managers from the selected clinical program. Approval from the Nursing and Medical Program Directors was sought before approaching individuals from the selected program.

**Interview schedule:** Questions were based on a theoretical framework for the process of change [3] and included details of the characteristics of the external environment; organisation; potential adopters; decisions; implementation strategies; barriers and enablers; degree of implementation; degree of practice change; patient, practitioner, system and economic outcomes; and respondents reflections on the current system. The full interview schedule is available [4].

**Data collection:** Interviews were approximately 1 hour long and were conducted in the interviewee's office or suitable meeting room. Interviews were not taped or transcribed but detailed notes were taken. Two CCE staff members attended, one as interviewer and one as note taker.

**Respondent validation:** Drafts were sent to the interviewees for clarification, comment and/or amendment as required.

**Analysis:** Final interview notes were collated and organised in MS Word and Excel using the elements of the scanning taxonomy. Emergent themes were identified by framework analysis.

**Response rate:** Representatives of 13 of the 14 committees, all 5 APU managers and 9 clinical managers participated. One committee Chair did not respond to the invitation for interview; due to lack of time no representative of this committee was interviewed. A surgical sub-specialty department head was unable to attend their interview and was replaced by a medical sub-specialty department head who was available at short notice.

**Representativeness of sample:** Almost all eligible committees and all eligible APUs were represented. The clinical managers represented Program Directors, Department Heads, Unit/Ward Managers and ancillary services; medical (n=4), nursing (n=4) and quality management (n=1) staff; in a range of sub-specialties across multiple campuses.

**Structured workshop with clinical decision-makers from a large diagnostic service**

**Aim:** To capture the actual process of capital equipment purchasing and identify how an ideal process for this decision-making might differ from current practice.

**Inclusion criteria:** Clinical managers involved in decisions regarding purchase or new or replacement equipment.

**Sampling:** Purposive sampling was used. A large multi-campus diagnostic service was selected based on their use of equipment and the interest in the project expressed by the Director.

**Approach:** The Director and Research Director of the department identified 18 suitable participants representing all health professional groups, all campuses and most units within the service. Personalised email invitations were sent by the Executive Director of Medical Services and Quality.

**Design:** An experienced facilitator from CCE who had no involvement in the SHARE project developed and delivered the workshop. A presentation on the background of the project and its relevance to the workshop was made by a SHARE project team member. Two other project team members were present to assist with logistics and note taking. The session was run over 1½ hours in the departmental seminar room. Five domains were identified a priori: how do we get an idea; what is the process (application, approval, feedback, who, timing); is it a good idea; is it the best idea; and monitoring and

evaluation.

**Data collection:** Using a nominal group technique, participants were asked to describe the ideal process for purchasing large capital equipment. Responses were collected on 'sticky-notes'. This method was repeated to identify gaps in the current process and included prioritisation of key areas for improvement.

**Respondent validation:** A workshop report was provided to participants for comment.

**Analysis:** Responses on the 'sticky notes' and additional workshop notes were collated and organised in MS Word and Excel using the domains identified a priori. Emergent themes were identified by framework analysis.

**Response rate:** 17 of the 18 invitees attended. An additional staff member from a clinical area not represented on the invitation list was included at the commencement of the workshop.

**Representativeness of sample:** Participants represented all campuses, sub-specialties and health professionals (medicine, nursing, allied health, technical, quality improvement, business management, research) within the department.

**Document analysis**

**Aim:** To provide evidence for the stated positions and methods of administration of decision-making systems and processes for resource allocation at Monash Health and the state health department.

**Inclusion criteria:** Documents that guided decision-making or implementation of resource allocation decisions

**Identification:** Documents were identified by key informants and searches within the Monash Health Policy and Procedure database.

**Documents included:** 1) State government: Victorian Government Purchasing Guidelines, Medical Equipment Asset Management Framework, Targeted Equipment Replacement Program and Health Purchasing Victoria Product Management Guidelines. 2) Monash Health: Purchasing Policy, Purchasing Policy Guidelines, Authority Delegation Schedule, Code of Conduct, Conflict of Interest Protocol, Guidelines for management of Gifts and Benefits, Terms of Reference for committees that make resource allocation decisions, Application forms, Business case templates, Requisition forms and checklists.

**Data collection:** Documents were retrieved or sourced online. Data were extracted based on a theoretical framework for the process of change [3].

**Collation:** Findings were collated and organised in MS Word and Excel using the elements of the theoretical framework.

**Consultation with purchasing and procurement staff**

**Aim:** To clarify purchasing and procurement processes; present proposals for change and identify additional opportunities; and discuss feasibility of proposals considered, implementation and evaluation.

**Participants:** Managers of Procurement, Clinical Purchasing and Health Technology Services; representative of SHARE Steering Committee; project team members.

**Design:** Agenda including points for discussion; decisions made by consensus; documentation of discussion, decisions and actions in minutes.

## 2. Guideline and protocol development

**Development of new Policy and Procedure Framework (PPF)**

**PPF project:** Not part of SHARE, separate project contemporaneously undertaken by members of SHARE project team: included rapid review of the literature; search for existing PPF frameworks; communication with Australian health agencies regarding standard definitions for policies, procedures, protocols and guidelines; consultation with Monash Health staff; review of local documents, processes and structures; comparison with practice in other health services.

**SHARE component:** Introduction of a prompt in the instructions to document developers to consider whether any current practices could be discontinued, and a requirement that a systematic review process was followed and a checklist recording the steps undertaken in document development were also included.

## 3. Proactive use of published research

**Development of catalogue of disinvestment opportunities**

**Literature searches**: Searches were conducted in known sources of high quality evidence using terms to identify health technologies and clinical practices with evidence of harm, lack of effect, and lack of cost-effectiveness

- Systematic reviews and Health Technology Assessments from Cochrane Database of Systematic Reviews, UK National Institute for Health and Clinical Excellence (NICE), Australian Safety and Efficacy Register of New Interventional Procedures-Surgical (ASERNIPS)
- Alerts, recalls and bulletins from Australian Therapeutic Goods Administration, Australian National Prescribing Service and Australia New Zealand Horizon Scanning Network
- Resources that specifically identified evidence-based targets for disinvestment such as NICE Optimal Practice Reviews and South Birmingham Primary Care Trust Not Routinely Funded List

**Opportunistic capture**: Potential disinvestment projects were proposed by SHARE Steering Committee, Technology/Clinical Practice Committee, project team and clinicians. Sources of ideas included conferences, journals articles, email bulletins and awareness of practice elsewhere. Claims regarding suitability for disinvestment were validated from the research literature by the project team.

**Development of taxonomy:** Classifications were based on existing definitions from the National Library of Medicine Medical Subject Headings (MeSH) [5]; International Statistical Classification of Diseases and Related Health Problems, Tenth Revision, Australian Modification (ICD-10-AM) [6]; McMaster Evidence Updates [7]; and Academy Health Glossary of Terms Commonly Used in Health Care [8]. When suitable definitions were not available, additional classifications were created and defined to meet Monash Health needs [9].

**Development of catalogue:** Details of 184 disinvestment opportunities were stored in an MS Excel spreadsheet using classifications from the taxonomy.

## 4. Proactive use of local data

**Consultation**

**Participants and Aims**

- Director of Clinical Information Management to identify opportunities and methods for accessing and using data to drive disinvestment decision-making.
- Director of Quality to discuss use of data in quality initiatives.
- Representatives of the 13 committees that make organisation-wide decisions regarding allocation of resources (noted above) to identify their ongoing and intermittent needs from local data.

**Design:** Informal individual discussions with SHARE project team members; the discussions with the committee representatives were conducted subsequent to the data gathering interviews noted above.

**Structured interviews with representatives from departments collecting, maintaining and sharing data related to TCPs**

**Aim:** To identify current sources of data at Monash Health and the processes involved.

**Inclusion criteria:** Managers of departments involved in collection, storage and use of data.

**Sampling:** Purposive sampling was used initially: departments were identified by the Head of Clinical Information Management and a concept paper on knowledge transfer at Monash Health. Snowball sample was used subsequently by asking respondents if they were aware of others. Representatives of 10 relevant departments were invited to participate (Clinical Information Management, Health Information Systems, Pharmacy, Pathology, Diagnostic Imaging, Research Directorate, Infection Control, Infectious Diseases and the Clinical Audit and Clinical Risk groups within the Quality Unit).

**Approach:** Email invitations were sent to the heads of the departments identified.

**Interview schedule:** Questions were designed to identify the data available, methods of collection and storage, utilisation in decision-making, internal and external reporting, other forms of dissemination, strengths and weaknesses of the current system and opportunities for improvement. The interview schedule is available [10].

**Data collection:** Interviews approximately 1 hour long were conducted by one CCE staff member and audio taped.

**Collation:** Interview data were collated in MS Excel using the elements of the interview schedule.

**Response rate:** All 10 invitees participated

**Representativeness of sample:** A broad range of settings were included.

## 5. Economic approaches to priority setting

**Literature review**

**Aim:** To identify examples of economic approaches to priority setting relevant to resource allocation for TCPs, decision-making criteria, and challenges of priority setting for disinvestment.

**Search terms:** Combinations of the terms priority, prioritisation, priority setting, priority criteria and health technology.

**Sources:** Medical databases (Ovid Medline, Cochrane Library, Cinahl), the internet (via the Google search engine), and prospective searches of identified review articles (in the Web of Science online search engine). These methods were supplemented by snowball searching for additional relevant articles from reference lists.

**Inclusion criteria:** Review articles, English language publications.

**Data Collection and Analysis:** Results were screened by title and abstract to identify recent review articles. Information relevant to local health service decision-making was extracted.

**Search results:** Two documents met the inclusion criteria.

**Synthesis:** Information was summarised in a discussion paper for a workshop with the SHARE Steering Committee (Table S5).

**Consultation**

Expert advice was sought from the SHARE health economist throughout this project.

## 6. System Redesign

**Literature review**

**Aim:** To investigate system redesign examples and their applicability to resource allocation decision-making for TCPs at Monash Health.

**Search terms:** Combinations of the terms system redesign, restructure, modernisation, clinical process redesign, lean, design principles, health system, healthcare reform, transformation, system change, organisational redesign, technology, clinical practice, priorities and disinvestment. The search strategy was iterative with new terms added as they were identified.

**Sources:** Medical databases (Ovid Medline, Embase, CINAHL, Cochrane Library), the internet (via the Google search engine) and specific websites including the UK National Health Service, the UK National Institute for Health and Clinical Excellence (NICE) and the US Institute for Healthcare Improvement (IHI). Key references, authors, organisations and reports highlighted in the literature were also investigated.

**Inclusion criteria:** Any study design, report or document discussing system redesign relevant to resource allocation decision-making for TCPs within a local health service, English language publications.

**Data Collection and Analysis:** Results were screened by title and abstract. Information relevant to local health service decision-making was extracted. The information retrieved was analysed in themes relevant to the SHARE aims ie system redesign, disinvestment, TCPs and decision-making in healthcare.

**Search results:** 682 articles were retrieved and 42 met the inclusion criteria.

**Synthesis:** Information was summarised in a discussion paper for a workshop with the SHARE Steering Committee (Table S5).

**Structured interviews with staff experienced in system redesign**

**Aim:** To investigate system redesign examples and their applicability to resource allocation decision-making for TCPs at Monash Health.

**Participants:** Experts in system redesign were initially identified by the Director of Quality; snowball sampling by asking interviewees if they were aware of others working in this area identified additional participants. Directors or senior managers in the areas of Strategic Planning; Access, Innovation and Service Improvement; Acute Ambulatory Services; Chronic Disease Management; Service Improvement, Quality and Projects, Mental Health; Diagnostic Imaging; General Medicine Model of Care Redesign; and Clinical Performance and Service Reconfiguration participated.

**Interview schedule:** Interviewees were asked about system redesign, their understanding of disinvestment, decision-making on new and existing services, prioritisation of services, processes for monitoring and evaluation, and communication strategies. Interview schedule in Table S6 below.

**Data collection:** Interviews lasting between 45 and 60 minutes were conducted by one CCE staff member who took notes.

**Analysis:** Content analysis was undertaken to identify emergent themes.

**Response rate:** All 8 invitees participated.

**Representativeness of sample:** All interviewees had expertise and experience in system redesign. This was an illustrative sample, no attempt was made to comprehensively identify all potential respondents within the organisation.

## Table S3. Investigation of methods for prioritisation and decision-making at Monash Health

| |
|---|
| **Literature review**<br>**Aim:** To identify existing frameworks and tools for prioritisation relevant to resource allocation for TCPs.<br>**Search terms:** Scoping search using the terms prioritisation, priority setting, priority criteria and health technology.<br>**Sources:** Medical databases (Ovid Medline, Cochrane Library) and the internet (via the Google search engine).<br>**Inclusion criteria:** Frameworks and tools for prioritisation relevant to the local healthcare setting or systematic reviews on this topic, English language publications.<br>**Data Collection and Analysis:** Results were screened by title and abstract to identify frameworks and tools for prioritisation. Information relevant to local health service decision-making was extracted.<br>**Search results:** 7 documents met the inclusion criteria; these included guidelines, frameworks, tools, systematic reviews and an overview of international practice in prioritisation of new technologies.<br>**Synthesis:** Information from articles which met the inclusion criteria was summarised in a discussion paper for a workshop with the SHARE Steering Committee (Table S5). |
| **Consultation**<br>**Aims:** To document government and local decision-making requirements for purchase of new and replacement of existing capital equipment and identify current practice at Monash Health.<br>**Participants:** Director of Business Support Services and Manager of Health Technology Services.<br>**Design:** Informal individual discussions with members of the project team who took notes. |

## Table S4. Investigation of methods for implementation and evaluation at Monash Health

| |
|---|
| **Structured interviews with staff members who have experience in disinvestment projects**<br>**Aim:** To learn from previous experiences of disinvestment at Monash Health.<br>**Inclusion criteria:** Staff who had undertaken projects to remove, reduce or restrict current practices (the term 'disinvestment' was not used in Monash Health projects).<br>**Sampling:** Purposive and snowball sampling was used. Relevant projects were initially identified by members of the SHARE Steering Committee and interviewees in the committee review process noted above. A snowballing method was employed by asking participating project representatives if they knew of any other relevant projects. Nineteen potential projects were identified, 13 met the inclusion criteria.<br>**Approach:** Personalised email invitations from the project team were sent to project managers of 13 relevant projects. Project managers or Department/Unit Heads were sought as key contacts; however a representative of the project team was accepted when a senior staff member was unavailable.<br>**Interview schedule:** Questions were designed to explore project governance, use of routinely-collected hospital data, other local data and research evidence in the development and implementation of projects; barriers and enablers to successful project implementation; what staff would do again and what they would do differently. The full interview schedule is available [4].<br>**Data collection:** Interviews were approximately 1 hour long and were conducted in the interviewee's office or suitable meeting room. Interviews were not taped or transcribed but detailed notes were taken. Two CCE staff members attended, one as interviewer and one as note taker.<br>**Respondent validation:** Drafts were sent to the interviewees for clarification, comment and/or amendment as required.<br>**Analysis:** Final interview notes were collated and organised in MS Word and Excel using the elements of the scanning taxonomy. Emergent themes were identified by framework analysis.<br>**Response rate:** Representatives of 10 projects participated based on interviewee's and interviewer's availability<br>**Representativeness of sample:** The process was designed to be illustrative and did not seek to comprehensively identify all projects. A number of project topics across a range of clinical areas were included. |
| **Structured interviews with decision-makers**<br>**Aim:** To identify and document current processes for implementing and evaluating decisions and the factors that influence them.<br>**Participants:** 27 staff members authorised to make decisions on behalf of the organisation (previously described in Table S2).<br>**Interview schedule, data collection and analysis:** Questions regarding implementation and evaluation of decisions at Monash Health were a subset of the broader interviews which are reported in Table S2. |

## Table S5. Deliberative process

**Structured workshops with SHARE Steering Committee**

**Aims:** To review and refine draft proposals, frameworks and plans and make final decisions.

**Participants:** SHARE Steering Committee members including Executive Directors (Medical, Nursing, Support Services), Program Directors (Medical, Nursing, Allied Health, Pharmacy, Diagnostic Services), Committee chairs (Technology/Clinical Practice, Therapeutics, Human Research and Ethics, Clinical Ethics), Managers (Information Services, Clinical Information Services, Procurement, Biomedical Engineering, Research Services), Legal counsel and two Consumer representatives.

**Design**

- Provision of pre-reading materials and/or workshop presentation of background, issues to consider, draft proposals, etc
- Agenda including points for discussion and decisions required
- Decisions made by consensus
- Documentation of discussion, decisions and actions in minutes

## Table S6. Interview schedule System Redesign

*The following questions focus on system redesign specifically in relation to technologies and clinical practices (TCPs).*

*Our aim is to investigate system redesign examples and their applicability to TCP resource allocation decision making in Southern Health.*

**System redesign**

1. What is your understanding of system redesign?
   a) Why do system redesign? eg better use of existing resources
2. How do you identify the need for system redesign?
3. What are some examples of system redesign within Southern Health? eg hospital-wide, department/s or ward/s.
   a) Where do you plan to implement system redesign in the future?
4. When do you plan and implement system redesign in the future? When is the 'right' or preferable time? May involve long-term projects organisation-wide or shorter-term individual projects within a department or ward.
   a) How do you decide which project is the priority?
5. Who is involved in the planning and implementation of system redesign? eg key stakeholders, executive management, staff and/or consumer representation
   a) How do you plan for system redesign? What resources are required? eg time, staff etc
6. How do you implement system redesign? What process is undertaken? eg an organisation-wide process of decision making and prioritisation or individual projects to implement change

**Disinvestment**

7. Are you familiar with the concept of disinvestment? What is your understanding of disinvestment? *Our understanding of disinvestment is: the process of (partially or completely) withdrawing health resources that are no longer considered to be safe, effective or cost-effective, and thus are not efficient health resource allocations.*
8. Is this concept actively considered in planning for system redesign in Southern Health? Specifically in relation to TCPs?

**Decision making**

9. How do you make decisions on planning for new services? Or changes in existing services?
   a) Assessment of how well new services will work / how well existing services work?
   b) Which new services to provide / spend money on?
   c) Which existing services to cease providing or enforce limitations/restrictions?
10. How do you prioritise allocation of new services? In particular, new TCPs?
11. What is your process for monitoring and evaluation of changes / collection of data?
12. How do you plan your communication strategy?
13. Any further thoughts or suggestions?

## References


1. Rumbold G, Allen K, Harris C. Disinvestment of technologies and clinical practices in health services: Conceptual and policy perspectives. Centre for Clinical Effectiveness, Southern Health 2008. Available from: http://arrow.monash.edu.au/hdl/1959.1/1218935. Accessed: October 2016
2. Harris C, Turner T, Wilkinson F. SEAchange: Guide to a pragmatic evidence-based approach to Sustainable, Effective and Appropriate change in health services. 2015. Available from: http://arrow.monash.edu.au/hdl/1959.1/1225377. Accessed: October 2016
3. Harris C, Green S, Ramsey W, Allen K, King R. Sustainability in Health care by Allocating Resources Effectively (SHARE) 1: Introducing an investigation of disinvestment in a local healthcare setting. BMC health services research. 2017;(Details TBA).
4. Harris C, Allen K, Waller C, Brooke V. Sustainability in Health care by Allocating Resources Effectively (SHARE) 3: Examining how resource allocation decisions are made, implemented and evaluated in a local healthcare setting BMC health services research. 2017;(Details TBA).
5. National Library of Medicine Medical Subject Headings (MeSH). https://www.nlm.nih.gov/mesh/. Accessed March 2016.
6. International Statistical Classification of Diseases and Related Health Problems, Tenth Revision, Australian Modification https://www.accd.net.au/Icd10.aspx. Accessed March 2016.
7. McMaster Evidence Updates. https://plus.mcmaster.ca/evidenceupdates/. Accessed March 2016.
8. Academy Health Glossary of Terms Commonly Used in Health Care. http://www.academyhealth.org/Training/content.cfm?ItemNumber=1657. Accessed March 2016.
9. Harris C, Garrubba M, Melder A, Voutier C, Waller C, King R et al. Sustainability in Health care by Allocating Resources Effectively (SHARE) 8: Developing, implementing and evaluating an Evidence Dissemination Service in a local healthcare setting. BMC health services research. 2017;(Details TBA).
